# Using machine‐learning methods to identify early‐life predictors of 11‐year language outcome

**DOI:** 10.1111/jcpp.13733

**Published:** 2022-12-07

**Authors:** Loretta Gasparini, Daisy A. Shepherd, Edith L. Bavin, Patricia Eadie, Sheena Reilly, Angela T. Morgan, Melissa Wake

**Affiliations:** ^1^ Murdoch Children’s Research Institute Parkville VIC Australia; ^2^ Department of Paediatrics The University of Melbourne Parkville VIC Australia; ^3^ School of Psychology and Public Health La Trobe University Bundoora VIC Australia; ^4^ Melbourne Graduate School of Education The University of Melbourne Parkville VIC Australia; ^5^ Menzies Health Institute Queensland Griffith University Gold Coast QLD Australia; ^6^ Department of Audiology and Speech Pathology The University of Melbourne Parkville VIC Australia; ^7^ Royal Children’s Hospital Melbourne Parkville VIC Australia; ^8^ Liggins Institute The University of Auckland Auckland New Zealand

**Keywords:** Language development, language disorders, sensitivity and specificity, longitudinal studies, machine learning

## Abstract

**Background:**

Language is foundational for neurodevelopment and quality of life, but an estimated 10% of children have a language disorder at age 5. Many children shift between classifications of typical and low language if assessed at multiple times in the early years, making it difficult to identify which children will have persisting difficulties and benefit most from support. This study aims to identify a parsimonious set of preschool indicators that predict language outcomes in late childhood, using data from the population‐based Early Language in Victoria Study (*n* = 839).

**Methods:**

Parents completed surveys about their children at ages 8, 12, 24, and 36 months. At 11 years, children were assessed using the Clinical Evaluation of Language Fundamentals 4th Edition (CELF‐4). We used random forests to identify which of the 1990 parent‐reported questions best predict children's 11‐year language outcome (CELF‐4 score ≤81 representing low language) and used SuperLearner to estimate the accuracy of the constrained sets of questions.

**Results:**

At 24 months, seven predictors relating to vocabulary, symbolic play, pragmatics and behavior yielded 73% sensitivity (95% CI: 57, 85) and 77% specificity (95% CI: 74, 80) for predicting low language at 11 years. [Corrections made on 5 May 2023, after first online publication: In the preceding sentence ‘motor skills’ has been corrected to ‘behavior’ in this version.] At 36 months, 7 predictors relating to morphosyntax, vocabulary, parent–child interactions, and parental stress yielded 75% sensitivity (95% CI: 58, 88) and 85% specificity (95% CI: 81, 87). Measures at 8 and 12 months yielded unsatisfactory accuracy.

**Conclusions:**

We identified two short sets of questions that predict language outcomes at age 11 with fair accuracy. Future research should seek to replicate results in a separate cohort.

## Introduction

Lifelong language skills are foundational for good socio‐behavioral, academic, employment, and quality of life outcomes (Conti‐Ramsden & Durkin, 2012; Eadie et al., 2018, 2021; Yew & O'Kearney, 2013). Language Disorder is defined as language difficulties impacting everyday functioning (Bishop et al., 2017) which affects an estimated 10% of children aged 5 (Norbury et al., 2016). Two‐thirds of these cases cannot be attributed to any specific condition or environmental factor (Norbury et al., 2016). Ideally, we could identify children across the whole population who will have persisting language difficulties from an early age, enabling us to provide targeted support while avoiding over‐servicing. This goal is complicated, however, by children shifting between classifications of typical and low language if assessed at multiple times in early childhood (approximately 6% of all children if assessed between 4 and 11 years, McKean, Wraith, et al., 2017), which yields a high error rate in identifying the estimated 10% of all children who will have persisting difficulties and most benefit from intervention. To best utilize the limited available resources to support children who will have persisting difficulties, we need early measures that accurately predict Language Disorder in later childhood.

Failing to punctually identify children at risk for persisting Language Disorder also hinders early intervention research. Currently, it is difficult to test the effectiveness of early language interventions because many recruited children catch up spontaneously, which drowns out the interventions' impact for the children who would otherwise have persisting difficulties (Wake et al., 2011). Accurately identifying Language Disorder in the preschool years will enable more precise recruitment into intervention programs, helping us to provide children with the language support they need to thrive.

Known predictors of poor language outcome observable before 3 years of age include: delayed gesture production, limited receptive and/or expressive vocabulary size, impaired syntactic development including absence of two‐word combinations, family history of Language Disorder, low socioeconomic status (SES), and poorer quality and quantity of communicative interactions (Sansavini et al., 2021). While there is growing consensus on predictors for language outcomes, the accuracy of language screening tools remains inconclusive as replications are sparse, most studies have assessed accuracy over short timeframes (1–2 years and within the preschool years) and their accuracy tends to attenuate with age (Wallace et al., 2015).

Prioritizing only items that uniquely contribute to predicting language outcome could lead to accurate, time‐efficient instruments suited to public health use. This approach draws upon other health areas that have identified short sets of questions that clinicians can readily collect via patient report or history (e.g., for 5‐year mortality in adults, Ganna & Ingelsson, 2015). Rudolph and Leonard (2016) asked parents retrospectively when their child was aged 4–7 years at what age their child started combining words. This question alone yielded 55% sensitivity and 93% specificity for classifying Language Disorder cases (at ages 4–7). Sensitivity improved (71%) but specificity decreased (84%) when they added family history of a Speech/Language Disorder and maternal education to the predictive model. Another study found that measures of vocabulary size, grammatical complexity and semantic network structure (semantic relatedness of vocabulary items the child is reported to know) collected between 16 and 30 months yielded >90% accuracy for classifying children with language or reading disorder (based on parental, school record, or clinician report between 4 and 7 years; Borovsky, Thal, & Leonard, 2021). Vocabulary size and semantic network structure measures however, require prolonged assessments such as administration of the entire MacArthur‐Bates Communicative Development Inventories (Words and Sentences, CDI‐WS) vocabulary checklist (680 items; Fenson et al., 2006) which limits the feasibility of implementing these measures at a population‐based level. By contrast, measures of grammatical complexity can be quick to administer and would be less burdensome for parents to complete if found to have adequate accuracy alone.

Another challenge in accurately predicting persisting Language Disorder is that salient predictors vary with age (Bishop et al., 2017). For a given set of predictors, we would want to identify the optimal age of data collection for best predictive accuracy. For instance, there is little value in collecting measures of morphosyntax at ages when they show floor effects across the population. Furthermore, as children encounter various health and education services in the early years, we would want various sets of predictors available at different child ages.

The Early Language in Victoria Study (ELVS) is a population‐based cohort study tracking language and communication from infancy to adolescence (Reilly et al., 2018). One study reporting on the ELVS data identified seven questions on the 12‐month parent survey that accounted for school‐entry language outcome with “fair” accuracy (Area Under the receiving operating characteristic Curve, AUC, of 0.73; McKean et al., 2016). These items related to gesture, vocabulary, family history of language/literacy difficulties, maternal education, SES, and parent–child interactions. Later ELVS studies dichotomized the expressive vocabulary CDI‐WS score at 2 years at a standardized score below the 10th percentile and found it did not reliably predict 7‐ (McKean, Reilly, et al., 2017) or 11‐year (Eadie et al., 2021) language. ELVS collected early morphosyntax measures at 2‐ and 3‐years of age via the CDI‐WS and MacArthur‐Bates Communicative Development Inventory ‐ Third Edition (CDI‐III, Dale, 2007), but these morphosyntax items are yet to be analyzed for their predictive accuracy.

The first aim of this study is to estimate the individual importance of a range of parent‐reported items (individual questions) collected in infancy for predicting low language outcome in late childhood (11 years). Note that “importance” is used as a technical term here, see Aim 1: Individual variable importance for how it is defined and calculated. The second aim is to use the results from the first aim along with knowledge of previous literature on language development, to identify a parsimonious set of parent‐reported items that could be used at various infant and toddler ages to accurately predict low language outcome at 11 years (see Outcome for how this is operationalized).

## Methods

We preregistered the statistical analysis methods on Open Science Framework (OSF) on 02/02/2022 at https://osf.io/fpdzk/ prior to data access by the first author who conducted the analysis. We published and timestamped protocol amendments and all Supporting information on the OSF repository. Ethical approval was obtained from the Royal Children's Hospital (23018, 27078, 82096) and La Trobe University Human Ethics Committee (03‐32). Parents provided written, informed consent.

### Sample

The Early Language in Victoria Study (ELVS) is a prospective longitudinal cohort study which recruited a community cohort of 1910 infants aged 7.5–10 months in six metropolitan areas of Melbourne, Victoria. Metropolitan areas were selected to represent all SES strata. Participants were recruited via the Maternal and Child Health Service (82% uptake rate), hearing screening visits and press advertising. The participants have been assessed in 11 waves from ages 8 months to 13 years (Reilly et al., 2018).

Participants were excluded from joining ELVS if at recruitment (when they were 8 months of age) the Maternal and Child Health Nurse identified them as having a disability likely to interfere with their language development, including developmental delay, cerebral palsy, a syndrome, intellectual or physical disability, cleft lip or palate, vision or hearing impairment. Children were also excluded if their parents did not understand English sufficiently to complete questionnaires designed for grade 6 level. As we are interested in predicting low language outcome for any child, we included participants with any co‐occurring conditions that did not preclude them from participating in ELVS (e.g., Autism, speech disorder). The primary analysis included all ELVS participants with data available at wave 10 (11 years old, *n* = 839, 44.6% of baseline participants).

### Predictors

At waves 1–4 (ages 8, 12, 24, 36 months), parents of ELVS participants completed surveys relating to their child, the family and home environment. The predictor measures in this study comprise all multiple‐choice or numerical items collected in these waves. Appendix S1 summarizes all constructs and instruments administered and describes our data cleaning and exclusion practices. Our analysis included 1990 variables (182 at 8 months, 644 at 12 months, 916 at 24 months, 248 at 36 months).

### Outcome

The 11‐year language outcome was measured using the Clinical Evaluation of Language Fundamentals, Fourth Edition, Australian version (CELF‐4, Semel, Wiig, & Secord, 2006), a standardized tool that yields a core language score with a mean of 100 and standard deviation (*SD*) of 15. The CELF‐4 is used as a diagnostic tool for Language Disorder and a core score below 77.5 was found to have 100% sensitivity and 89% specificity for diagnosing Language Disorder (Pearson Education, 2008). Because information on the everyday functional impact of children's language abilities is also needed for a diagnosis of Language Disorder (Bishop et al., 2016), we consider a low CELF‐4 score as a potential indicator of Language Disorder. We operationalized the dichotomous outcome as “low” (≥1.5 *SD* below the mean) and “typical” (<1.5 *SD* below the mean) language. We selected this cut‐off as a balance between maximizing diagnostic accuracy (according to Pearson Education, 2008) and ensuring the low language group had sufficient participants for the models to successfully run.

### Statistical methods

We conducted statistical analyses in RStudio (R Core Team, 2020; RStudio Team, 2020) and code is available on the OSF repository. We provide more details of the statistical methods in Appendices S2 (random forests) and S3 (SuperLearner).

#### Aim 1: Individual variable importance

We ran random forests, a tree‐based machine‐learning method, separately by data collection wave to estimate the importance of all 1990 variables. Of the 642 variables with missing values, we excluded variables with >50% missingness (172 variables). For the remainder, we imputed missing values using the rfImpute command in the *randomForest* package (Liaw & Wiener, 2002). We repeated the imputation process 300 times to produce 300 imputed datasets. For each imputed dataset, we computed a random forest with 500 unbiased conditional inference trees using the package *party* (Hothorn, 2005; Strobl, Boulesteix, Zeileis, & Hothorn, 2007; Strobl, Hothorn, & Zeileis, 2009). Variable importance was estimated using conditional permutation importance without replacement (Strobl et al., 2009), where larger variable importance values indicate a closer relationship between the predictor and outcome. We averaged variable importance values across the 300 imputed datasets. We also completed a complete case sensitivity analysis (see Sensitivity analyses).

#### Aim 2: Predictor set selection

We used the estimated variable importance values from Aim 1 to help select a constrained set of predictors. We evaluated variables with the highest variable importance values for the extent to which they accord with previous research on predictors of language outcome (e.g., Sansavini et al., 2021; Zambrana, Pons, Eadie, & Ystrom, 2014) and would likely be easy to understand and answer by any caregiver. For each data collection wave, we selected eight questions to include in the models, which approximates 1 min administration time.

We used SuperLearner with 10‐fold cross‐validation to estimate the predictive accuracy of our sets of predictors in predicting our language outcome. SuperLearner utilizes various prediction algorithms and weights them according to their accuracy in a new single algorithm (van der Laan, Polley, & Hubbard, 2007). We used a complete case analysis to estimate predictive accuracy. In our SuperLearner algorithm we included the following base learners: elastic net regression, random forests, extreme gradient boosting, Bayesian additive regression trees, multivariate adaptive regression splines (see Appendix S3 for details).

For each set of predictors (8, 12, 24 and 36 months), we started with eight measures and iteratively excluded one variable, by lowest variable importance, to determine the optimal number of variables to include in our final set. For each final set of predictors, we calculated AUC, sensitivity and specificity with 95% confidence intervals (CIs). For the purposes of interpreting results we consider sensitivity and specificity >70% as “fair” and > 80% “good” classification accuracy (we decided these thresholds in our preregistration prior to starting the analysis, based on previous studies using ELVS data, Eadie et al., 2021; McKean et al., 2016). We also ran this analysis cumulatively by data collection wave.

#### Sensitivity analyses

As a sensitivity analysis, we repeated the Aim 2 analysis (predictor set selection) with the cut‐off of low and typical language being 1.25 and 2 *SD*s below the mean CELF‐4 score. We also repeated the Aim 2 analysis with 7‐year language (wave 8) as the outcome (1.25, 1.5, and 2 *SD*s below the mean cut‐offs), to see if our results were robust to including a larger sample as fewer participants had been lost to attrition by age 7. This included all participants for whom a CELF‐4 core language score was available at 7 years (*n* = 1,208, 63.2% of baseline participants).

We ran a complete case analysis of the Aim 1 analysis (random forests) to see whether our results can be attributed to patterns in the real data and not imputed data.

#### Additional analyses

We ran univariate logistic regressions on all variables included in the final predictor sets, as well as a constrained number of predictors which we expected from previous literature might have a relationship with the outcome, to check that the effects were in the directions we expect and to allow comparison with other literature.

We assessed the classification accuracy of the preferred predictor sets in the subgroup of participants where an additional language to English was spoken at home or to the child at any ages between 8 and 36 months.

In a separate study we will use data from Growing Up in Australia: The Longitudinal Study of Australian Children (LSAC, Sanson & Johnstone, 2004) to replicate the current study (Gasparini, Shepherd, Wang, Wake, & Morgan, 2022). Therefore, we ran SuperLearner models using only those variables ranked highly in the Aim 1 analysis that LSAC also collected.

## Results

### Participant characteristics

At 11 years, 1,071 individuals were lost to follow‐up and 839 were retained. Comparing the two groups shows that the retained participants were overrepresented with girls, families where English was the only main language spoken to the child, mothers with higher education levels, and higher advantage based on postcode (Appendix S4).

The included sample had a mean 11‐year CELF‐4 score of 100.6 (*SD* = 12.89, range: 40.0–129.0). The 1.5 *SD* below the mean cut‐off was at a raw score of 81 and resulted in 791 (94.3%) participants in the typical language group and 48 (5.7%) in the low language group. A raw score of 81 corresponds to 1.25 *SD* below the mean using the population‐based standardized scores, showing that our sample has on average better language skills than the wider population.

### Aim 1: Individual variable importance

All variables from each data collection wave were ranked by their estimated variable importance and the top 10 variables from each wave are reported in Table 1.

**Table 1:** Top 10 predictors from each wave and their estimated variable importance

Question	Levels or scale	Source	Variable importance
Wave 1 (8 months)			
What was the last year of school your partner completed?^a^	Year 10 or less Year 11 Year 12	ELVS	0.0000636
How difficult do you think your life is at present?^a^	No problems or stresses Few problems or stresses Some problems or stresses Many problems or stresses Too many problems or stresses	ATP	0.0000505
Child's age	Numeric (months)	ELVS	0.0000173
I help my child learn by talking and showing him/her new things^a^	Not very often Sometimes Often	BITS	0.0000140
Does your child throw a ball?^a^	Yes No	CDI‐WG	0.0000067
Does your child blow raspberries?^a^	Yes No	ELVS	0.0000055
When you are not paying attention to your child, does he/she try to get your attention?^a^	Not Yet Sometimes Often	CSBS	0.0000043
Smacks lips in a “yum yum” gesture to indicate when something tastes good	Not Yet Sometimes Often	CDI‐WG	0.0000042
Caregiver age^a^	Numeric (years)	ELVS	0.0000039
About how long were you pregnant with this baby?^a^	Numeric (weeks)	ELVS	0.0000019
Wave 2 (12 months)			
I am this child's:^b^	Natural/biological father Stepfather Other	ELVS	0.0007905
Does your child string sounds together?^a^	Not Yet Sometimes Often	CSBS	0.0001533
Does your child use sounds or words to get attention or help?^a^	Not Yet Sometimes Often	CSBS	0.0000678
In the last 2 weeks, about how often did you feel so sad nothing would cheer you up?^a^	None of the time A little of the time Some of the time Most of the time All of the time	Kessler‐K6	0.0000673
About how many of the following objects does your child use appropriately: cup, bottle, bowl, spoon, comb or brush, toothbrush, washcloth, ball, toy vehicle, toy telephone?^a^	None 1–2 3–4 5–8 Over 8	CSBS	0.0000210
Child understands (and says) “give”^a^	Understands Understands and says Neither	CDI‐WG	0.0000149
Child understands (and says) “don't”^a^	Understands Understands and says Neither	CDI‐WG	0.0000104
Child understands “give me a kiss”	Yes No	CDI‐WG	0.0000055
Child understands (and says) “drink”	Understands Understands and says Neither	CDI‐WG	0.0000046
Does your child sing?^a^	Yes No	CDI‐WG	0.0000029
Wave 3 (24 months)			
About how many of the following objects does your child use appropriately: cup, bottle, bowl, spoon, comb or brush, toothbrush, washcloth, ball, toy vehicle, toy telephone?^a^	3–4 5–8 Over 8	CSBS	0.0005823
Does your child use sounds or words to get attention or help?^a^	Not yet Sometimes Often	CSBS	0.0001384
Do you have any concerns about how your child behaves?^a^	No A little Yes	PEDS	0.0001242
Child says “spoon”^a^	Says Does not say	CDI‐WS	0.0000440
About how many different words or phrases does your child understand without gestures?^a^	1–3 4–10 11–30 Over 30	CSBS	0.0000421
Child says “face”^a^	Says Does not say	CDI‐WS	0.0000282
Child says “quack quack”^a^	Says Does not say	CDI‐WS	0.0000265
Does your child talk about objects that are not present?^b^	Not yet Sometimes Often	CDI‐WS	0.0000254
Child says “teddy bear”	Says Does not say	CDI‐WS	0.0000199
Child says “bubbles”	Says Does not say	CDI‐WS	0.0000196
Wave 4 (36 months)			
Mark the sentence that sounds most like the way your child talks:^a^	This dolly big This dolly big and this dolly little	CDI‐III	0.0005135
Has your child begun to combine words yet?^a^	Not yet Sometimes Often	CDI‐III	0.0004320
Child says “circle”^a^	Says Not yet	CDI‐III	.0002802
Child says “accident”^a^	Says Not yet	CDI‐III	0.0001717
Child says “kangaroo”	Says Not yet	CDI‐III	0.0000811
Child says “forget/forgot”^a^	Says Not yet	CDI‐III	0.0000633
I play with my child and show him/her things^a^	Not very often Sometimes Often	BITS	0.0000524
How do you think you are coping?^a^	Not at all A little Fairly well Very well Extremely well	ATP	0.0000422
Can your child answer questions?^b^	Yes No	CDI‐III	0.0000345
Child says “hurry”	Says Not yet	CDI‐III	0.0000317

^a^ Variable was included in the final/preferred model.

^b^ Variable was included in SuperLearner (Aim 2) analysis but excluded from the final/preferred model.

[Corrections made on 5 May 2023, after first online publication: For Wave 3 in Table 1, ‘Do you have any concerns about how your child uses his/her arms and legs?’ has been corrected to ‘Do you have any concerns about how your child behaves?’ in this version.]

### Aim 2: Predictor set selection

Here we justify why we deviated from the order of variable importance values when choosing which predictors to include in the SuperLearner models. We explain which predictors we included in the final sets.

In wave 1 (8 months) we excluded “Smacks lips in a “yum yum” gesture to indicate when something tastes good.” This was because we already included “Does your child blow raspberries?” and we considered both to target oromotor skills. This meant we could include gestational age at birth, which is known to impact language development (Rudolph, 2017). We also excluded child's age, which is not useful as a predictor. The most accurate predictor set included all eight of the variables included in the SuperLearner models.

In wave 2 (12 months) we deprioritized questions about understanding the phrases/words “give me a kiss” and “drink”, as the vocabulary items “give” and “don't” (also verbs and often used as an imperative) were already included. Instead, we included “Does your child sing?”, capturing speech production, pragmatic and more general cognitive skills. Although the question “I am this child's natural/biological father, stepfather or other” had the highest variable importance score, it had more missing data than the other variables and we considered that clinicians might not feel comfortable asking this in all contexts. Therefore, we dropped this variable first (instead of in order of variable importance) and found that the most accurate predictor set excluded this variable and included the remaining 7.

For wave 3 (24 months) we were satisfied with the top 8 in order of variable importance. The most accurate predictor set included the top 7 variables.

In wave 4 (36 months) we excluded the vocabulary item “kangaroo” as it might disadvantage recent migrant families to Australia, and we already included the multisyllabic nouns “circle” and “accident.”. This meant we could include “Can your child answer questions?” which captures more general language use. However, the most accurate predictor set excluded this final question and included just the top 7 variables.

Table 1 indicates the 8 variables we included in the SuperLearner models and the variables we selected for the final predictor set. Table 2 presents the AUC, sensitivity and specificity of each of these final predictor sets, by data collection wave. The SuperLearner model is the weighted average of all prediction algorithms, while Discrete is the single algorithm with the best predictive ability. The SuperLearner model was the most accurate for the 8‐, 12‐ and 24‐month sets and the discrete model (extreme gradient boosting, see Appendix S3 for more details) was more accurate for the 36‐month set. The 24‐ and 36‐month sets reached “fair” accuracy levels, while accuracy levels of the 8‐ and 12‐month sets were unsatisfactory. Combining the variables between data collection waves did not substantially improve accuracy (see results in Appendix S5). Table 3 shows the final predictor sets that yielded fair accuracy (24 and 36 months).

**Table 2:** Estimated AUC, sensitivity and specificity (95% CIs) by wave obtained using SuperLearner. “N” indicates number of participants included after missing data were removed. “Model” indicates if SuperLearner or discrete was the most accurate model

	*n*	Model	AUC	Sensitivity	Specificity
Wave 1 (8 months)	740	SuperLearner	0.64 (0.54, 0.74)	0.65 (0.47, 0.80)	0.62 (0.59, 0.66)
Wave 2 (12 months)	822	SuperLearner	0.63 (0.54, 0.72)	0.54 (0.39, 0.69)	0.64 (0.60, 0.67)
Wave 3 (24 months)	804	SuperLearner	0.77 (0.68, 0.86)	0.73 (0.57, 0.85)	0.77 (0.74, 0.80)
Wave 4 (36 months)	739	Discrete	0.79 (0.70, 0.89)	0.75 (0.58, 0.88)	0.84 (0.81, 0.87)

**Table 3:** Final predictor sets that yielded fair accuracy (at ages 24 and 36 months)

Question	Source
Wave 3 (24 months)	
About how many of the following objects does your child use appropriately: cup, bottle, bowl, spoon, comb or brush, toothbrush, washcloth, ball, toy vehicle, toy telephone?	CSBS
Does your child use sounds or words to get attention or help?	CSBS
Do you have any concerns about how your child behaves?	PEDS
Child says “spoon”	CDI‐WS
About how many different words or phrases does your child understand without gestures?	CSBS
Child says “face”	CDI‐WS
Child says “quack quack”	CDI‐WS
Wave 4 (36 months)	
Mark the sentence that sounds most like the way your child talks: “This dolly big” or “This dolly big and this dolly little”	CDI‐III
Has your child begun to combine words yet?	CDI‐III
Child says “circle”	CDI‐III
Child says “accident”	CDI‐III
Child says “forget/forgot”	CDI‐III
I play with my child and show him/her things	BITS
How do you think you are coping?	ATP

[Corrections made on 5 May 2023, after first online publication: For Wave 3 (24 months) in Table 3, ‘Do you have any concerns about how your child uses his/her arms and legs?’ has been corrected to ‘Do you have any concerns about how your child behaves?’ in this version.]

### Sensitivity analyses

Sensitivity analyses of the SuperLearner models using alternative outcome measures yielded the same interpretation of results (see Appendix S6).

The random forests complete case analysis resulted in substantial data loss (205 participants with missing data at 8 months, 512 at 12 months, 512 at 24 months, and 583 at 36 months) and in mostly different variables being ranked highly by variable importance (Appendix S7).

### Additional analyses

Univariate logistic regression results showed that effects were all in the expected directions according to previous literature: in general, a skill or supportive environment is associated with being in the typical language group at 11 years (Appendix S8).

The multilingual subgroup analysis had very small numbers of participants (*n* = 39) and low precision so results cannot be considered meaningful (Appendix S9).

The analyses using variables included in LSAC yielded similar results to our primary analysis: unsatisfactory (AUC < 70%) accuracy for variables collected at 8 and 12 months and fair (AUC > 70%) for those collected at 24 and 36 months (Appendix S10).

## Discussion

We have identified two short (< 1 min) sets of questions that can be asked at 24 or 36 months with “fair” accuracy (≥73% sensitivity and specificity) for predicting which children will have low language skills at 11 years (CELF‐4 core language score ≤ 81). These measures relate to children's vocabulary, morphosyntax, symbolic play, pragmatic skills, behavior, parent–child interactions and parental stress. [Corrections made on 5 May 2023, after first online publication: In the preceding sentence, ‘pragmatic‐ and motor‐skills’ has been corrected to ‘pragmatic skills, behavior’ in this version.] Measures at 8 and 12 months of age yielded unsatisfactory accuracy in predicting the dichotomous 11‐year language outcome.

We expected the 8‐ and 12‐month measures to yield higher accuracy based on a previous ELVS study that identified seven 12‐month predictors yielding >70% accuracy for 5‐year language (McKean et al., 2016). Considering that both used ELVS data, the differing results might be attributed to us selecting a later language outcome (11 years). Our results suggest that parent‐reported measures collected at or before 12 months are inadequate for predicting persisting language difficulties. This may be because children's communication skills are less meaningful at this stage for predicting later outcomes. Alternatively, it may be more difficult for parents to assess their child's preverbal comprehension abilities than their later productive skills, making parent‐reported measures less suitable at such early ages.

As expected, the final predictor sets at 24 and 36 months related to children's sentence complexity, vocabulary, language use, parent–child interactions, and parental stress (related to SES), according with previous research (Sansavini et al., 2021). Children's use of objects and their general behavior were also predictive at 24 months, warranting further consideration of symbolic play and behavior for predicting language outcome. [Corrections made on 5 May 2023, after first online publication: In the preceding sentence, ‘arms and legs’ has been corrected to ‘general behavior’, ‘motor skills’ has been corrected to ‘behavior’ and the reference to Gonzalez et al. (2019) regarding motor skills has been removed here and in the References section.] The 24‐ and 36‐month predictor sets yielded lower accuracy than Borovsky et al. (2021), who showed that vocabulary size, semantic network structure and morphosyntax measures yielded >90% accuracy. Some methodological differences may explain the difference in results, including that our study had a later outcome age, larger sample size and all participants were assessed using the same standardized language test (*n* = 839 at 11 years, and *n* = 1,208 at 7 years tested using the CELF‐4, while Borovsky et al., 2021, had *n* = 476 at 4–7 years assessed using one of parental, school record or clinician report). While our predictor sets have lower predictive accuracy, they are also shorter and so would be much quicker to complete and to score, making it more feasible to implement widespread (if results replicate and generalize in our next study, Gasparini et al., 2022).

### Strengths and limitations

The Early Language in Victoria Study recruited participants from across whole communities, spanning a range of SES strata. This sampling approach is a strength to our aim of identifying language disorders across the whole population. In contrast, many language studies recruit through speech/language services, which can bias samples toward participants with more severe impairments and better‐serviced communities.

This study's only exclusion criterion was of children with developmental disability or morbidity so significant that it was already evident at age 8 months (the age of recruitment to ELVS). While it could be of interest to determine how predictive values might change if children with conditions (e.g., Autism, ADHD) were excluded, this does not reflect real‐world clinical services because diagnosis of such conditions in Australia still usually occurs not only after the age of our baseline, but often after the ages at which all our predictor sets were collected for this study.

Another strength is how we operationalized language outcome. Our primary language outcome was at 11 years of age, striving to capture children with language difficulties persisting throughout childhood. The language outcome was measured using the CELF‐4, a standardized tool that can be used for diagnostic purposes. Our results remained robust when we operationalized the language outcome at a different age (7 years) when the sample size was larger, and when we set alternative cut‐off points for dichotomizing the language outcome. This strengthens the evidence that our predictors can classify children who will have persisting language difficulties.

Our use of machine‐learning methods offers many strengths. Random forests is non‐parametric, robust to correlated predictors and can manage many variables with relatively few observations (Tagliamonte & Baayen, 2012). Hence, we could reduce a range of predictors to a manageable set. SuperLearner minimizes the effects of arbitrary methodological choices when selecting a prediction algorithm and setting parameters by running and evaluating multiple options (McNamara, Zisser, Beevers, & Shumake, 2022). Both random forests and SuperLearner implement cross‐validation, which avoids overfitting (Lever, Krzywinski, & Altman, 2016).

A limitation is the wide 95% CIs of sensitivity estimates due to the low population prevalence of Language Disorder. A larger sample size would yield more precise sensitivity. Moreover, attrition over the 10 years from baseline to 11‐year outcome resulted in a more socio‐economically privileged sample compared with the group lost to follow‐up. Due to the sample size and not being representative of the wider population, our multilingual subgroup analysis failed to yield meaningful results.

The complete case sensitivity analysis using random forests identified a largely different set of predictors than the primary analysis. This could suggest the primary analysis contains artifacts due to imputed values or that the complete case analysis is biased and less precise due to high levels of missing data. We are comfortable interpreting results based on the analysis with imputed values because we averaged results across 300 different imputations, minimizing the effects of individual imputed values, and the SuperLearner analysis used complete case analysis and yielded satisfactory results for the 24‐ and 36‐month predictor sets. Multiple imputation using data adaptive approaches like SuperLearner remains an open statistical challenge, as it is difficult to define an imputation model that is compatible with the prediction model (Dashti et al., 2021). Hence, we used a complete case analysis to estimate the accuracy of predictor sets, but this might have introduced bias or reduced precision by excluding participants with missing data.

### Future directions

Next, we will test the accuracy of the predictor sets we identified in a separate population‐based cohort to assess how our results generalize to the wider population (Gasparini et al., 2022). The sets of predictors should also be assessed for their efficacy and reliability when collected in real‐world conditions.

We opted for a population‐wide approach in identifying predictors, but future work could focus on identifying precise predictors in subgroups. Namely, a future study should evaluate the accuracy of the measures we identified on a larger group of multilingual children. Multilingual children are at risk both of over‐ and under‐diagnosis of Language Disorder (Grimm & Schulz, 2014) and so would especially benefit from accurate early identification and support. Future work could also focus on predicting which children with a given condition (e.g., Autism) are at greater risk of poor language outcomes.

Parent‐reported measures are a useful resource because they currently yield comparable accuracy to direct assessments in the early years (Wallace et al., 2015), are inexpensive and low burden. However, technological advances may improve precision by enabling accurate, direct measures of predictors that can be collected on a home device and automatically coded (e.g., Chang et al., 2021; identifying Autism). Polygenic risk scores may also allow us to identify genetic variance largely unaccounted for by phenotypic measures and improve accuracy (Mountford, Braden, Newbury, & Morgan, 2022).

## Conclusion

We have identified two short sets of questions that can be asked at 24 or 36 months in under 1 min with “fair” accuracy (≥73% sensitivity and specificity) for predicting which children will have low language skills at 11 years. The next step is to replicate our findings in a separate population‐based cohort to assess whether these sets of predictors have adequate accuracy for predicting language outcome in the wider population.

## Supporting information


**Appendix S1.** Predictor measures ages 8–36 months.
**Table S1.** Constructs collected by ELVS, instrument and number of items per wave included in the current analysis.
**Appendix S2.** Random forests.
**Figure S1.** Schematic of how the dataset initially includes missing values for the variable “combines words”.
**Figure S2.** The rows are randomly selected from the full dataset into a bootstrapped dataset, where replacement is allowed.
**Figure S3.** A decision tree taking a subset of columns and splitting by levels or the most suitable cut‐off and determining from the bootstrapped dataset what is the most common outcome at each node.
**Figure S4.** The previous steps are repeated hundreds of times, each time creating a new bootstrapped dataset and a new decision tree.
**Figure S5.** All the decision trees are assessed for the outcome using the out‐of‐bag data (data that were not included in the bootstrapped dataset used to build the trees).
**Figure S6.** Comparison of the “real” data, with actual values of the variable “combines words” and the “permuted” data, with random values of the variable.
**Figure S7.** Each variable is given multiple variable importance values based on different datasets with different imputed values.
**Appendix S3.** SuperLearner.
**Appendix S4.** Participant characteristics.
**Table S2.** Baseline characteristics of participants lost and retained at 11 years; values are % except where indicated as mean (*SD*).
**Appendix S5.** Combining variables by wave.
**Table S3.** Results of SuperLearner models separately and cumulatively by wave.
**Appendix S6.** Sensitivity analysis using alternative outcome measures.
**Table S4.** Summary of CELF‐4 scores at 11 and 7 years, and the number of participants in typical and low language groups at different cut‐off scores, before (“total”) and after (waves 1–4) missing data were removed.
**Table S5.** AUC of SuperLearner models with alternative outcome ages (11 or 7 years) and cut‐offs (1.25, 1.5 or 2 *SD* below the mean).
**Appendix S7.** Complete case analysis of random forests analysis.
**Table S6.** Number of participants classified as having “normal” (CELF‐4 raw score >81 at 11 years) or “low” (≤81) language included in each wave of the random forests primary and complete case analysis.
**Table S7.** Top 10 variables per wave ranked by variable importance in the complete case analysis.
**Appendix S8.** Univariate analysis.
**Table S8.** Odds ratio and 95% CI of having low language outcome at 11 years (CELF‐4 raw score < 81).
**Table S9.** Odds ratios and 95% CIs of selected variables based on previous literature for predicting 11‐year language outcome.
**Appendix S9.** Multilingual subgroup analysis.
**Table S10.** SuperLearner accuracy (AUC), sensitivity and specificity by wave in the multilingual subgroup of participants.
**Table S11.** SuperLearner accuracy (AUC) by wave in the multilingual subgroup of participants with the 7‐year language outcome.
**Appendix S10.** Exploratory analysis of predictors mapping to LSAC variables.
**Table S12.** The 8 variables in the best‐fitting model from ELVS waves 1–2 that correspond to LSAC wave 1 variables.
**Table S13.** The 6 variables in the best‐fitting model from ELVS waves 3–4 that correspond to LSAC wave 2 variables.
**Table S14.** The 11 variables in the best‐fitting model from ELVS waves 1–4 that correspond to LSAC wave 1‐2 variables.
**Table S15.** Accuracy (95% CIs) of the best‐fitting models using ELVS variables that correspond to LSAC variables.
