# Supplementary material for: Using machine‐learning methods to identify early‐life predictors of 11‐year language outcome
Source: J Child Psychol Psychiatry. 2022 Dec 7;64(8):1242–52. doi: 10.1111/jcpp.13733 (PMC10952842; doi:10.1111/jcpp.13733)
Supplement: Supplementary file 1 — Appendix S1. Predictor measures ages 8–36 months. Table S1. Constructs collected by ELVS, instrument and number of items per wave included in the current analysis. Appendix S2. Random forests. Figure S1. Schematic of how the dataset initially includes missing values for the variable “combines words”. Figure S2. The rows are randomly selected from the full dataset into a bootstrapped dataset, where replacement is allowed. Figure S3. A decision tree taking a subset of columns and splitting by levels or the most suitable cut‐off and determining from the bootstrapped dataset what is the most common outcome at each node. Figure S4. The previous steps are repeated hundreds of times, each time creating a new bootstrapped dataset and a new decision tree. Figure S5. All the decision trees are assessed for the outcome using the out‐of‐bag data (data that were not included in the bootstrapped dataset used to build the trees). Figure S6. Comparison of the “real” data, with actual values of the variable “combines words” and the “permuted” data, with random values of the variable. Figure S7. Each variable is given multiple variable importance values based on different datasets with different imputed values. Appendix S3. SuperLearner. Appendix S4. Participant characteristics. Table S2. Baseline characteristics of participants lost and retained at 11 years; values are % except where indicated as mean (SD). Appendix S5. Combining variables by wave. Table S3. Results of SuperLearner models separately and cumulatively by wave. Appendix S6. Sensitivity analysis using alternative outcome measures. Table S4. Summary of CELF‐4 scores at 11 and 7 years, and the number of participants in typical and low language groups at different cut‐off scores, before (“total”) and after (waves 1–4) missing data were removed. Table S5. AUC of SuperLearner models with alternative outcome ages (11 or 7 years) and cut‐offs (1.25, 1.5 or 2 SD below the mean). Appendix S7. Complete case analysis of random for [file JCPP-64-1242-s001.docx]

**Using machine-learning methods to identify early-life predictors of 11-year language outcome**

OSF repository: <https://osf.io/fpdzk/>

Please cite as: Gasparini, L., Shepherd, D. A., Bavin, E. L., Eadie, P., Reilly, S., Morgan, A. T., & Wake, M. (2022). Using machine-learning methods to identify early-life predictors of 11-year language outcome. *Journal of Child Psychology & Psychiatry*. <https://doi.org/10.1111/jcpp.13733>

*Appendix S1: Predictor measures ages 8-36 months*

Table S1 summarizes the measures included in the analysis. Collected variables were based on a biodevelopmental framework of early life factors (Shonkoff, 2010) and validated instruments of children’s early language, communication and behavior, maternal psychological distress and parents’ vocabulary as well as questions designed by the ELVS investigators and indices of socio-economic status.

1. *Constructs collected by ELVS, instrument and number of items per wave included in the current analysis*

| **Constructs** | **Pertains to** | **Instrument** | **Number of items** | | | |
| --- | --- | --- | --- | --- | --- | --- |
|  |  |  | **W1** | **W2** | **W3** | **W4** |
| Aboriginal and/or Torres Strait Islander status | M, Pa | ELVS | 2 |  |  |  |
| Age | C, M, F | [ELVS](https://lifecourse.melbournechildrens.com/cohorts/elvs/birth/sdv-birth-wave-1/) | 2 | 1 | 1 | 1 |
| Antibiotics for ear infection | C | [ELVS](https://lifecourse.melbournechildrens.com/cohorts/elvs/birth/sdv-birth-wave-1/) | 2 | 2 | 2 | 2 |
| Birth order | C | [ELVS](https://lifecourse.melbournechildrens.com/cohorts/elvs/birth/sdv-birth-wave-1/) | 1 |  |  |  |
| Birth weight | C | [ELVS](https://lifecourse.melbournechildrens.com/cohorts/elvs/birth/sdv-birth-wave-1/) | 1 |  |  |  |
| Communication and social skills | C | CSBS-DP-ITC | 24 | 24 | 24 |  |
| Communication and social skills | C | [CDI-WG](https://lifecourse.melbournechildrens.com/cohorts/elvs/language-literacy/cdi-macarthur-bates-communicative-development-inventories-wave-1-2003-2004-8-months-parent-report/) | 35 | 64 |  |  |
| Coping | M | ATP | 1 | 1 | 1 | 1 |
| Country of birth | M, Pa | [ELVS](https://lifecourse.melbournechildrens.com/cohorts/elvs/birth/sdv-birth-wave-1/) | 4 |  |  |  |
| Development | C | PEDS | 7 | 7 | 7 | 8 |
| Eating behaviors | C | ELVS | 6 | 6 |  |  |
| Ear infections | C | [ELVS](https://lifecourse.melbournechildrens.com/cohorts/elvs/birth/sdv-birth-wave-1/) | 2 | 2 | 2 | 2 |
| Early life childcare/ education | C | [ELVS](https://lifecourse.melbournechildrens.com/cohorts/elvs/birth/sdv-birth-wave-1/) | 3 | 3 | 3 | 5 |
| Education level | M, Pa | [ELVS](https://lifecourse.melbournechildrens.com/cohorts/elvs/birth/sdv-birth-wave-1/) | 14 |  |  |  |
| Family history of language or literacy problems | M, F | ELVS | 8 |  |  |  |
| Functioning | C | PedsQL-4.0 |  |  |  | 21 |
| Gestational age/prematurity | C | [ELVS](https://lifecourse.melbournechildrens.com/cohorts/elvs/birth/sdv-birth-wave-1/) | 1 |  |  |  |
| Grammar | C | CDI-WS |  |  | 117 |  |
| Grammar | C | CDI-III |  |  |  | 25 |
| Hearing | C | [ELVS](https://lifecourse.melbournechildrens.com/cohorts/elvs/birth/sdv-birth-wave-1/) | 3 | 1 | 1 | 1 |
| Hearing tests | C | [ELVS](https://lifecourse.melbournechildrens.com/cohorts/elvs/birth/sdv-birth-wave-1/) | 3 | 3 | 3 | 3 |
| Income/employment | M, Pa | [ELVS](https://lifecourse.melbournechildrens.com/cohorts/elvs/birth/sdv-birth-wave-1/) | 2 |  |  |  |
| Intimate/romantic relations | M | ELVS |  | 2 | 2 | 2 |
| Language spoken at home | C | [ELVS](https://lifecourse.melbournechildrens.com/cohorts/elvs/birth/sdv-birth-wave-1/) | 3 |  | 3 | 3 |
| Living arrangements/family composition | C | [ELVS](https://lifecourse.melbournechildrens.com/cohorts/elvs/birth/sdv-birth-wave-1/) | 15 | 1 | 1 | 1 |
| Marital status | C | [ELVS](https://lifecourse.melbournechildrens.com/cohorts/elvs/birth/sdv-birth-wave-1/) | 1 |  |  |  |
| Multiple births | C | [ELVS](https://lifecourse.melbournechildrens.com/cohorts/elvs/birth/sdv-birth-wave-1/) | 1 |  |  |  |
| Neonatal intensive/special care | C | [ELVS](https://lifecourse.melbournechildrens.com/cohorts/elvs/birth/sdv-birth-wave-1/) | 2 |  |  |  |
| Parental-child interactions | C | BITS | 10 | 10 | 10 | 10 |
| Reading/books | C | ELVS |  |  | 2 | 1 |
| Relationship to child | Pa |  |  | 1 |  | 1 |
| Sex | C | [ELVS](https://lifecourse.melbournechildrens.com/cohorts/elvs/birth/sdv-birth-wave-1/) | 1 |  |  |  |
| Socio-economic status | C | SEIFA | 5 |  |  |  |
| Speech | C | ELVS | 12 | 28 | 18 | 2 |
| Speech/language services utilization | C | ELVS |  | 7 | 5 | 7 |
| Stress | C | Kessler-K6 |  | 6 | 6 | 6 |
| Stress | M | ATP | 1 | 1 | 1 | 1 |
| Stuttering | C | ELVS |  |  |  | 1 |
| Sucking behavior | C | [ELVS](https://lifecourse.melbournechildrens.com/cohorts/elvs/birth/sdv-birth-wave-1/) | 6 | 6 | 6 |  |
| Temperament and behavior | C | [ATP](https://lifecourse.melbournechildrens.com/cohorts/elvs/behavioural-problems/atp-australian-temperament-project-wave-4/) | 1 | 15 | 15 | 40 |
| Tympanostomy tubes (grommets) | C | [ELVS](https://lifecourse.melbournechildrens.com/cohorts/elvs/birth/sdv-birth-wave-1/) | 1 | 1 | 1 | 1 |
| Vocabulary | C | [CDI-WG](https://lifecourse.melbournechildrens.com/cohorts/elvs/language-literacy/cdi-macarthur-bates-communicative-development-inventories-wave-1-2003-2004-8-months-parent-report/) |  | 430 |  |  |
| Vocabulary | C | CDI-WS |  |  | 679 |  |
| Vocabulary | C | CDI-III |  |  |  | 100 |
| Vocabulary | M | MHVS |  | 21 |  |  |

**C:** Index child; **M:** Mother; **F:** Father; **Pa:** Other parent; [**ATP**](https://lifecourse.melbournechildrens.com/cohorts/elvs/behavioural-problems/atp-australian-temperament-project-wave-4/)**:** Australian Temperament Project derived (Prior et al., 2000; Sewell et al., 1988); **BITS:** Brigance Parent-Child Interactions Scale (Glascoe & Brigance, 2002); **CDI-III**: MacArthur-Bates Communicative Development Inventories, Third Edition (Dale, 2007); **CDI-WG**: MacArthur-Bates Communicative Development Inventories (Words and Gestures) (Fenson et al., 1993); **CDI-WS**: MacArthur-Bates Communicative Development Inventories (Words and Sentences) (Fenson et al., 1993); **CSBS-DP-ITC**: Communication and Symbolic Behavior Scales Developmental Profile Infant-Toddler Checklist (Wetherby & Prizant, 2002); [**ELVS**](https://lifecourse.melbournechildrens.com/cohorts/elvs/birth/sdv-birth-wave-1/): ELVS-designed variable; **Kessler-K6**: The Kessler Psychological Distress Scale (K6 version) (Kessler & Mroczek, 1994); **MHVS**: Mill Hill Vocabulary Scale (Raven, 1997); **PEDS**: Parents' Evaluation of Developmental Status (Glascoe, 1999); **PedsQL-4.0**: Pediatric Quality of Life Inventory – 4.0 Generic Core Scales (Varni et al., 2003); **SEIFA**: Socio-Economic Indexes for Areas (Australian Bureau of Statistics, 2018)

For the current study we excluded open-ended questions. To avoid convergence issues, we converted factors with more than 10 levels to a categorical or numerical response with fewer than 10 levels if meaningful, otherwise we excluded it.

We excluded variables with >50% missingness.

Where meaningful, we imputed values questions with high levels of missing data. For example, questions of the form “Has your child ever…” with a follow-up question “How many times?” had high data missingness, as anyone who answered “no” to the first question left the second question blank. Thus, we imputed values into the second question whereby if a responder answered “no” to the first question, the answer to the second question was imputed as “0” (if numerical) or a new level “never” (if categorical). For example, for the questions “In the last 12 months, has this child seen a doctor for an ear infection?” and “How many times?” if the answer to the first question was “no”, then we imputed the value “0” for the second question.

In contrast, if the follow-up question was “At what age?” we could not impute a meaningful value. For example, for the questions “Does this child regularly receive childcare?” and “From what age did your child start childcare?”, if the child has never received childcare, there is no meaningful numerical child age to impute into the second question. In these cases, we deleted the variable if there was >50% missingness. This was the case for the questions “From age did your child start childcare?” and “About how old was your child at the last hearing test?”.

At wave 1 we deleted factors relating to siblings’ speech, language or reading problems due to high missingness. At wave 2, 65 of mothers’ vocabulary items were excluded due to high missingness. All 86 of fathers’ vocabulary items were deleted due to high missingness.

*Appendix S2: Random forests*

Here we expand on the methods described in the *Aim 1: Individual variable importance* section of the main manuscript. In “Background” we explain the concept of random forests and provide helpful resources. In “Implementation” we provide further details on how we implemented random forests in our study.

**Background**

Random forests is a tree-based machine-learning algorithm used for classification and regression. It is suitable for investigating the role of a large number of variables, including by ranking them by order of importance, splitting continuous variables into meaningful binary partitions and identifying interactions (Breiman, 2001; Tagliamonte & Baayen, 2012). Due to the large number of variables we considered in our study (1990), we prefer this technique over traditional methods such as logistic regression, which would fail to converge.

For introductory videos on random forests see Körting (2014), Starmer (2018, 2020) and Udacity (2016). Explanations of random forests and how to implement them in R using the package party (Hothorn, 2005; Strobl et al., 2007, 2009) can be found in Tagliamonte and Baayen (2012) and de Aguiar et al. (2016).

We use Lucida Console font to show R packages, functions and parameters.

**Implementation**

The whole process works through the following steps:

1. Impute missing values
2. Create a bootstrapped dataset
3. Create a decision tree using the bootstrapped dataset
4. Repeat steps (2) and (3) hundreds of times
5. Classify all rows in test data using the decision trees
6. Estimate variable importance
7. Repeat steps (1)-(6) and average variable importance across databases with different imputed values

The images below depict a dataset fabricated for illustrative purposes using 5 participants (rows) and 3 variables (gestational age in weeks, understands “don’t” yes/no, combines words yes/no). In our analysis presented in the paper, these steps included all 851 participants and all variables from each wave of data.

- - 1. **Impute missing values**

We imputed missing values using the rfImpute function in the package randomForest (Liaw & Wiener, 2002). We set this to 10 iterations (iter=10) of 300 trees (ntree=300). We reduced this from our initial plan of 100 iterations of 2000 trees due to prohibitively long computational times. The function imputes values by estimating a participant’s proximity to other participants based on other variables where values are not missing. It then imputes the most common level or weighted average of the missing values accordingly to similar participants.

1. *Schematic of how the dataset initially includes missing values for the variable “combines words”. The imputation function identifies similar rows based on other variables (color-coded with green or purple respectively in the second dataset) and imputes values accordingly*


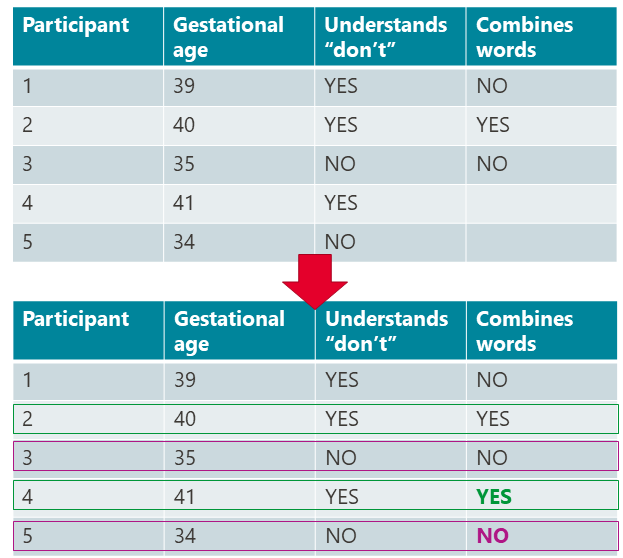


- - 1. **Create a bootstrapped dataset**

The cforest function from the package party (Hothorn, 2005; Strobl et al., 2007, 2009) runs the following steps 2-5.

The function randomly selects rows (participants) from the original sample with replacement (duplicated rows) allowed. This process of randomly subsetting the data is called “bagging” and occurs because we set the parameter within control = cforest_unbiased as mtry=NULL. This means that the accuracy of the tree’s predictions will be evaluated by comparing its predictions (based on in-bag observations) for the out-of-bag data with the out-of-bag observed values (in step 5). This is a process of internal cross-validation and avoids overfitting the model to noise in the data.

1. *The rows are randomly selected from the full dataset into a bootstrapped dataset, where replacement is allowed*


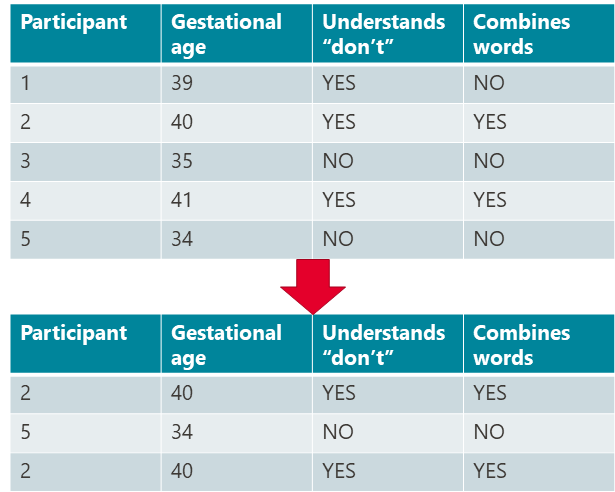


- - 1. **Create a decision tree using the bootstrapped dataset**

The function creates a decision tree using the bootstrapped dataset, using only a random subset of columns (predictors) at each step. It splits categorical variables by levels and continuous variables by the cut-off that best partitions the outcome, then uses the bootstrapped dataset to determine the most common outcome based on those levels or values of the predictors.

This decision tree is called a “base learner”. The cforest function uses “unbiased conditional inference trees” as their base learners. “Unbiased” is set by adding the parameter control = cforest_unbiased, which avoids having a bias towards continuous variables or categorical variables with more levels (see Strobl et al., 2009).

1. *A decision tree taking a subset of columns and splitting by levels or the most suitable cut-off and determining from the bootstrapped dataset what is the most common outcome at each node*


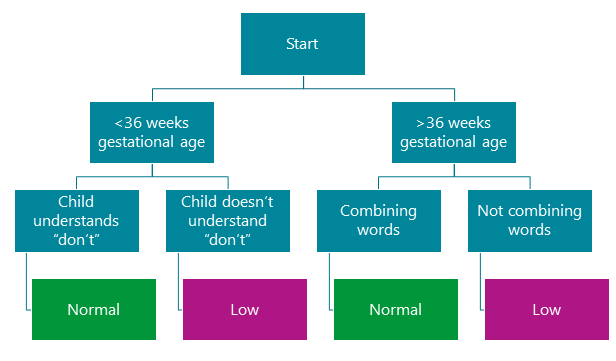


- - 1. **Repeat steps (2) and (3) 500 times**

Within the argument control = cforest_unbiased we can set further parameters, including ntree=500, meaning 500 trees are created. This creates a *forest* of *trees.*

1. *The previous steps are repeated hundreds of times, each time creating a new bootstrapped dataset and a new decision tree*


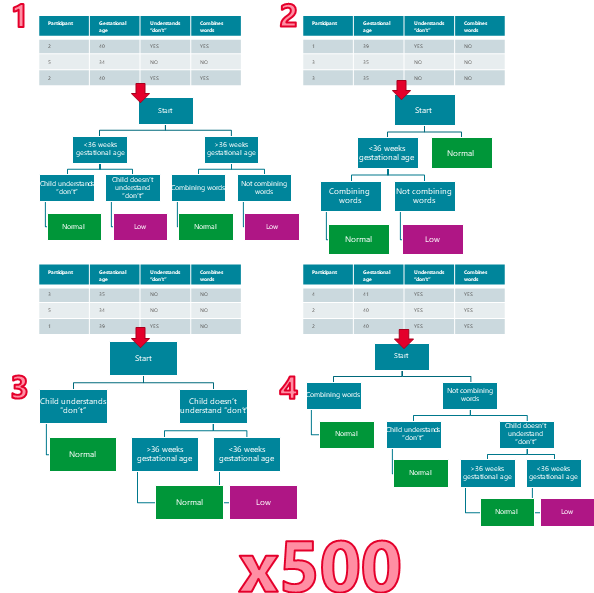


- - 1. **Classify all rows in test data using the decision trees**

The algorithm then takes the test or “out-of-bag” data (rows that weren’t included in the bootstrapped dataset in step (2)) and asks all trees what the outcome is (“low” or “normal” language). It comes up with one classification per row, by counting the votes of all the trees. It can then calculate classification accuracy by comparing imputed classifications from the random forest and actual classifications.

1. *All the decision trees are assessed for the outcome using the out-of-bag data (data that were not included in the bootstrapped dataset used to build the trees)*


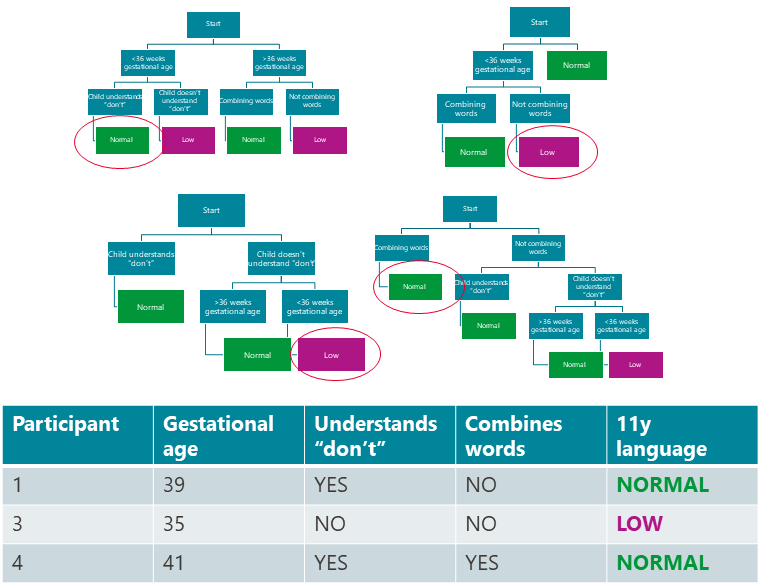


- - 1. **Estimate variable importance**

Next, we run the function varimp to estimate the importance of each variable in predicting the outcome, or specifically, to calculate “conditional permutation importance without replacement”. The function does estimates variable importance by randomly permuting different levels or values of the variable into each row. This means there is no correlation between the predictor variable and the outcome in the permuted data. If the predictor is truly associated with the outcome, then the model using the real data should be more accurate than the model with the randomly permuted data. Based on how much better the real data performs, a variable importance measure is calculated for each variable, where larger values indicate a closer relationship between the predictor and outcome. Zero and negative values indicate no/a small relationship with the outcome.

“Conditional” permutation importance is set with the parameter conditional=TRUE. This considers correlations between predictors that create spurious correlations between the outcome and an unrelated variable and attenuates importance measures accordingly (see Strobl et al., 2009).

1. *Comparison of the “real” data, with actual values of the variable “combines words” and the “permuted” data, with random values of the variable. Here the real dataset is found to be better at classifying the outcome, and so the variable “combines words” is given a relatively high variable importance value*


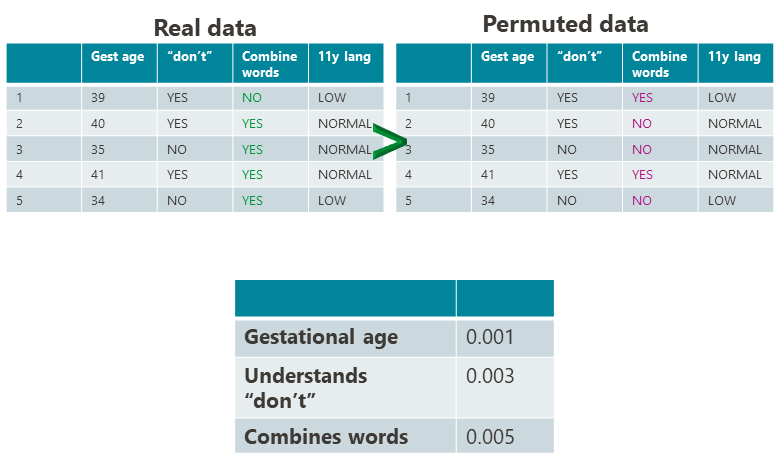


- - 1. **Repeat steps 1-6 and average variable importance across databases with different imputed values**

We conducted steps 1-6 in a for loop, so they repeat 300 times, creating 300 databases with different imputed values to run steps 2-6 on. This minimises artefacts in the results due to imputed values. For each variable we get 300 variable importance measures and then we calculate the average importance metric for each variable.

Then we can inspect and plot the average variable importance measures to see which variables are the best predictors of the outcome.

1. *Each variable is given multiple variable importance values based on different datasets with different imputed values. We impute all variable importance values to obtain an average for each predictor variable.*


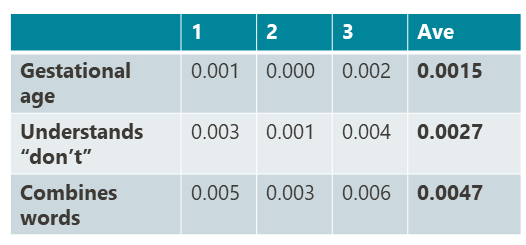


*Appendix S3: SuperLearner*

Here we expand on the methods described in the *Aim 2: Predictor set selection* section of the main manuscript. In “Background” we explain the concept of SuperLearner and provide helpful resources. In “Implementation” we provide further details on how we implemented SuperLearner in our study

**Background**

SuperLearner is an ensemble algorithm that utilizes various prediction algorithms (e.g. logistic regression, random forests), evaluates how well each performed and then weights each one accordingly in a new single prediction algorithm that is expected to perform at least as well as any of the individual methods (van der Laan et al., 2007).

For an illustrated introduction, see Hoffman (2019). For the SuperLearner package vignette with code on how to implement it in R, see Kennedy (2017). For another tutorial on running the SuperLearner package see (Gremmell, 2018).

To learn about many of the individual algorithms (“base learners”) you can include in your SuperLearner, see Starmer (2022) for introductory videos and Boehmke and Greenwell (2020) for running in R.

We use Lucida Console font to show R packages, functions and parameters.

**Implementation**

We used complete case analysis to estimate the accuracy of predictors as it is difficult in SuperLearner to define an imputation model that is compatible with the prediction model (Dashti et al., 2021).

The whole process works through the following steps:

1. Select base learners (individual algorithms) to include in the SuperLearner
2. Running the SuperLearner function:
   1. Split data into 10 blocks for cross-validation
   2. Train the base learners of 9 on the 10 blocks of data
   3. Obtain predictions from each base learner for the 1 block of test data
   4. Repeat steps (2)-(3) until all 10 blocks have served as test data
   5. Weight all base learners based on which maximize accuracy (AUC)
   6. Obtain predictions from the full data set for each base learner
   7. Weight predictions based on step 5 to obtain final predictions for each row
3. Evaluate the predictions by extracting the SuperLearner’s predictions and comparing to the actual outcomes. Calculate equal cut-point sensitivity and specificity with 95% CIs
4. **Select base learners to include in the SuperLearner**

You can see all possible wrappers (the functions for running the base learner in SuperLearner) by running listWrappers().

We used the following wrappers:

- Elastic net regression, including lasso and ridge (glmnet) (Friedman et al., 2010; Hastie et al., 2021). Lasso and ridge fit generalized models (like traditional regression) but apply a penalty that attenuates the slope and identifies the best fitting model based on the least amount of variance (least likely to overfit). Lasso and ridge use different equations to apply this penalty and elastic net regression combines both lasso and ridge into a hybrid.
- Conditional tree forest (cforest) (Hothorn, 2005; Strobl et al., 2007, 2009). Random forests, for detail see Appendix S2.
- Extreme gradient boosting (xgboost) (Chen et al., n.d., 2022; Chen & Guestrin, 2016). A tree-based method (as is random forests) but creates smaller decision trees than random forests. New trees are based on previous trees’ errors and evaluate the gain in further splitting leaves.
- Bayesian additive regression trees (bartMachine) (Kapelner & Bleich, 2016; Mamun, 2021). Uses weak learner decision trees like gradient boosting but uses a regularization prior to avoid overfitting.
- Multivariate adaptive regression splines (earth) (Milborrow, 2021a, 2021b). Captures non-linear relationships by assessing cut-points (“knots”) in the data and fits separate linear models within all cut-point ranges. Using cross-validation, the number of knots is pruned to find the optimal number and avoid overfitting.

We attempted to also include logistic regression (glm), k nearest neighbors (kernelKnn) (Mouselimis, 2021), support vector machines (svm) (Meyer et al., n.d.) and neural networks (nnet) (Venables & Ripley, 2002) but models failed to converge, so we excluded these.

We selected these algorithms as they are widely used in predicting binary outcomes, utilize a variety of methods (regression- and tree-based, parametric and non-parametric) and we found they had relatively efficient computation times.

If the default settings of the SuperLearner wrapper differed from the default settings of the R package, we created a new wrapper with the R package default settings and included both in the SuperLearner function.

1. **Running the SuperLearner function…**

Using the function CV.SuperLearner. We set family = binomial() as the outcome is dichotomous.

- 1. **Split data into 10 blocks for cross-validation**

We set the number of cross-validation blocks using cvControl = list(V = 10)

- 1. **Train the base learners of 9 on the 10 blocks of data**

All the algorithms we want to include are listed in the CV.SuperLearner function under SL.library

- 1. **Obtain predictions from each base learner for the 1 block of test data**
  2. **Repeat steps (2)-(3) until all 10 blocks have served as test data**
  3. **Weight all base learners based on which maximize accuracy (AUC)**

This is done by adding all base learners into a “meta-learner” model. We tell the meta-learner algorithm to maximize AUC as the way of evaluating the base learners by setting method = "method.AUC".

- 1. **Obtain predictions from the full data set for each base learner**

This happens by inputting the whole dataset into each of the base learners and generating predictions based on each base learner.

- 1. **Weight predictions based on step (5) to obtain final predictions for each row**

1. **Evaluate the predictions by extracting the SuperLearner’s predictions and comparing to the actual outcomes. Calculate equal cut-point sensitivity and specificity with 95% CIs**

This step uses the packages cvAUC (LeDell et al., 2014), caret (Kuhn, 2021) and epiR (Stevenson & Sergeant, 2021). We extract the SuperLearner predictions by running cv_sl$SL.predict, (where cv_sl is the name we gave our SuperLearner object) which is a vector of the estmated probability of each participant having “low” language. We use the function ci.cvAUC() to compare the predicted outcome with the observed language outcome along every probability cut-point and calculate the area under the receiving operating characteristic curve (AUC). Then we identify the probability cut-off that optimises both sensitivity and specificity. We run the function epi.tests() to calculate sensitivity and specificity with 95% confidence intervals.

We repeat the above using the Discrete model (by running cv_sl$discreteSL.predict). The SuperLearner model is the weighted average of all prediction algorithms, while Discrete is the single algorithm with the best predictive ability. If the discrete model is more accurate than the SuperLearner model, it is suitable to take the results of the discrete model (Phillips et al., 2022).

*Appendix S4: Participant characteristics*

For R code and output for the below results, go to *R_ELVS > ELVS-participant-characteristics.Rmd* on the OSF repository.

Table S2 shows that the retained participants were overrepresented with girls, English only being the main language spoken to the child, mothers with higher education levels, and higher advantage based on postcode.

1. *Baseline characteristics of participants lost and retained at 11 years; values are % except where indicated as mean (SD)*

|  | **Lost (n=1071)** | **Retained (n=839)** |
| --- | --- | --- |
| Female | 46.3 | 53.5 |
| Twin birth | 3.3 | 2.1 |
| Preterm birth (<36 weeks gestation) | 2.6 | 3.6 |
| Birth weight (kg), mean (SD) | 3.4 (0.5) | 3.4 (0.5) |
| Birth order |  |  |
| First | 49.9 | 50.4 |
| Second | 35.6 | 35.0 |
| Third | 11.4 | 12.5 |
| Fourth or later | 3.1 | 2.1 |
| Language other than English spoken to the child | 9.6 | 3.1 |
| Maternal education, last year of school completed |  |  |
| Year 10 or less | 12.2 | 5.9 |
| Year 11 | 14.7 | 13.3 |
| Maternal education, completed degree or postgraduate | 28.7 | 37.3 |
| Socio-Economic Indexes for Areas (SEIFA) Advantage-Disadvantage value by post code (Australian Bureau of Statistics, 2018), mean (SD) | 1026.8 (66.3) | 1043.4 (59.1) |
| Parent had speech, language or reading problems | 16.7 | 16.9 |
| Maternal age at baseline, mean (SD) | 31.31 (4.7) | 32.53 (4.2) |

*Appendix S5: Combining variables by wave*

For R code and output for the below results, go to *R_ELVS > SL > eleven-year15* on the OSF repository.

Table S3 shows the results of predictor sets individually and combined by wave. The SuperLearner model is the weighted average of all prediction algorithms, while Discrete is the single algorithm with the best predictive ability. If the discrete model is more accurate than the SuperLearner model, it is suitable to take the results of the discrete model (Phillips et al., 2022).

Combining variables between waves tended not to substantially improve model accuracy. Combining waves 1-3 yielded .79 (95% CI: [.71,.88]) accuracy, which was a slight improvement to .76 (95% CI: [.68,.86]) achieved at wave 3 alone. Combining waves 1-4 yielded .81 (95% CI: [0.71, 0.91]), which was a slight improvement to .79 (95% CI: [0.70, 0.88]) at wave 4 alone.

1. *Results of SuperLearner models separately and cumulatively by wave. Bold indicates the primary analysis reported in paper. Asterisk indicates where a slight improvement was achieved by combining waves compared to a single timepoint*

|  | AUC (95% CI) | |
| --- | --- | --- |
|  | SuperLearner | Discrete |
| Wave 1 (8 months) | **0.63 (0.54, 0.72)** | 0.59 (0.50, 0.69) |
| Wave 2 (12 months) | **0.63 (0.54, 0.72)** | 0.61 (0.52, 0.70) |
| Wave 3 (24 months) | **0.76 (0.68, 0.86)** | 0.74 (0.64, 0.83) |
| Wave 4 (36 months) | 0.76 (0.66, 0.87) | **0.79 (0.70, 0.88)** |
| Waves 1-2 | 0.64 (0.55, 0.73) | 0.66 (0.56, 0.75) |
| Waves 1-3 | 0.76 (0.68, 0.85) | 0.79 (0.71, 0.88)* |
| Waves 1-4 | 0.81 (0.71, 0.91)* | 0.76 (0.65, 0.87) |
| Waves 2-4 | 0.76 (0.66, 0.87) | 0.78 (0.69, 0.88) |
| Waves 3-4 | 0.71 (0.58, 0.84) | 0.79 (0.69, 0.89) |

*Appendix S6: Sensitivity analysis using alternative outcome measures*

For R code and output for the below results, go to *R_ELVS > SL > eleven-year2, eleven-year125, seven-year2, seven-year15, seven-year125* on the OSF repository.

Table S4 summarizes the CELF-4 scores at both outcome ages of 11 and 7 years and describes the number of participants in the typical and low language groups at each cut-off score. It also shows the number of participants included in each part of the analysis after we deleted cases of missing data.

Table S5 shows the results of all the models using alternative outcome measures. The SuperLearner model is the weighted average of all prediction algorithms, while Discrete is the single algorithm with the best predictive ability. If the discrete model is more accurate than the SuperLearner model, it is suitable to take the results of the discrete model (Phillips et al., 2022). The results in bold are the primary analysis reported in the paper (11-year language with cut-off of 1.5SD below the sample mean, i.e., a raw score of 81).

The results show that the 8- and 12-month models remain unsatisfactory in almost all cases (although the 8-month model reaches “fair”, >70%, levels of accuracy when the 2SD below the mean cut-off is used). The 24- and 36-month models are robust to any outcome measures and have “fair” (>70%) accuracy in all cases, and occasionally reach “good” (>80%) levels.

Accuracy tended to improve the lower the cut-off score. This suggests that the models are accurate at identifying more severe cases of low language ability, but that they are less accurate at identifying children who may be considered having borderline cases of language disorder.

The consistency of results at 7 and 11 years given the larger sample size at 7 years provides support that the results we see using the 11-year outcome is not an artefact of attrition in the sample between 7 and 11 years.

1. *Summary of CELF-4 scores at 11 and 7 years, and the number of participants in typical and low language groups at different cut-off scores, before (“total”) and after (waves 1-4) missing data were removed.*

| **Outcome age** | **n** | **Mean** | **SD** | **Range** | **Cut-off (SD below mean)** | **Raw score** | **N (%) in each group** | | |
| --- | --- | --- | --- | --- | --- | --- | --- | --- | --- |
|  |  |  |  |  |  |  | **Waves** | **Typical** | **Low** |
| **11y** | **839** | **100.6** | **12.89** | **40.0-129.0** | 1.25 | 84.53 | Total | 772 (92.01) | 67 (7.99) |
|  |  |  |  |  |  |  | Wave 1 | 688 (92.97) | 52 (7.01) |
|  |  |  |  |  |  |  | Wave 2 | 757 (92.09) | 65 (7.91) |
|  |  |  |  |  |  |  | Wave 3 | 742 (92.29) | 62 (7.71) |
|  |  |  |  |  |  |  | Wave 4 | 685 (92.69) | 54 (7.31) |
|  |  |  |  |  | **1.5** | **81.31** | **Total** | **791 (94.28)** | **48 (5.72)** |
|  |  |  |  |  |  |  | **Wave 1** | **703 (95.00)** | **37 (5.00)** |
|  |  |  |  |  |  |  | **Wave 2** | **776 (94.40)** | **46 (5.60)** |
|  |  |  |  |  |  |  | **Wave 3** | **760 (94.53)** | **44 (5.47)** |
|  |  |  |  |  |  |  | **Wave 4** | **703 (95.13)** | **36 (4.87)** |
|  |  |  |  |  | 2 | 74.86 | Total | 816 (97.26) | 23 (2.74) |
|  |  |  |  |  |  |  | Wave 1 | 722 (97.57) | 18 (2.43) |
|  |  |  |  |  |  |  | Wave 2 | 801 (97.45) | 21 (2.55) |
|  |  |  |  |  |  |  | Wave 3 | 783 (97.39) | 21 (2.61) |
|  |  |  |  |  |  |  | Wave 4 | 725 (98.11) | 14 (1.89) |
| 7y | 1208 | 97.63 | 13.40 | 40.0-134.0 | 1.25 | 80.88 | Total | 1104 (91.39) | 104 (8.61) |
|  |  |  |  |  |  |  | Wave 1 | 957 (92.46) | 78 (7.54) |
|  |  |  |  |  |  |  | Wave 2 | 1065 (91.73) | 96 (8.27) |
|  |  |  |  |  |  |  | Wave 3 | 1051 (91.95) | 92 (8.05) |
|  |  |  |  |  |  |  | Wave 4 | 939 (93.15) | 69 (6.85) |
|  |  |  |  |  | 1.5 | 77.53 | Total | 1133 (93.79) | 75 (6.21) |
|  |  |  |  |  |  |  | Wave 1 | 980 (94.69) | 55 (5.31) |
|  |  |  |  |  |  |  | Wave 2 | 1091 (93.97) | 70 (6.03) |
|  |  |  |  |  |  |  | Wave 3 | 1077 (94.23) | 66 (5.77) |
|  |  |  |  |  |  |  | Wave 4 | 963 (95.54) | 45 (4.46) |
|  |  |  |  |  | 2 | 70.83 | Total | 1169 (96.77) | 39 (3.23) |
|  |  |  |  |  |  |  | Wave 1 | 1006 (97.20) | 29 (2.80) |
|  |  |  |  |  |  |  | Wave 2 | 1125 (96.90) | 36 (3.10) |
|  |  |  |  |  |  |  | Wave 3 | 1110 (97.11) | 33 (2.89) |
|  |  |  |  |  |  |  | Wave 4 | 986 (97.82) | 22 (2.18) |

1. *AUC of SuperLearner models with alternative outcome ages (11 or 7 years) and cut-offs (1.25, 1.5 or 2SD below the mean). Bold indicates the primary analysis reported in paper*

| **Outcome age** | **Cut-off (raw)** | **Model** | **8 months** | **12 months** | **24 months** | **36 months** |
| --- | --- | --- | --- | --- | --- | --- |
| 11-year | 1.25 (84) | SuperLearner | 0.56 (0.48, 0.65) | 0.62 (0.54, 0.70) | 0.73 (0.65, 0.80) | 0.69 (0.60, 0.79) |
|  |  | Discrete | 0.55 (0.46, 0.64) | 0.60 (0.53, 0.68) | 0.72 (0.64, 0.80) | 0.72 (0.63, 0.80) |
|  | 1.5 (81) | SuperLearner | **0.64 (0.54, 0.74)** | **0.63 (0.54, 0.72)** | **0.77 (0.68, 0.86)** | 0.76 (0.66, 0.87) |
|  |  | Discrete | 0.59 (0.50, 0.69) | 0.61 (0.52, 0.70) | 0.74 (0.64, 0.83) | **0.79 (0.70, 0.89)** |
|  | 2 (74) | SuperLearner | 0.73 (0.63, 0.83) | 0.66 (0.55, 0.76) | 0.83 (0.71, 0.94) | 0.81 (0.64, 0.98) |
|  |  | Discrete | 0.66 (0.54, 0.78) | 0.65 (0.54, 0.76) | 0.85 (0.75, 0.95) | 0.80 (0.66, 0.93) |
| 7-year | 1.25 (80) | SuperLearner | 0.60 (0.54, 0.66) | 0.52 (0.45, 0.57) | 0.75 (0.70, 0.81) | 0.75 (0.70, 0.81) |
|  |  | Discrete | 0.57 (0.50, 0.63) | 0.50 (0.44, 0.56) | 0.79 (0.72, 0.85) | 0.80 (0.74, 0.86) |
|  | 1.5 (77) | SuperLearner | 0.57 (0.49, 0.65) | 0.53 (0.46, 0.60) | 0.75 (0.68, 0.82) | 0.77 (0.69, 0.85) |
|  |  | Discrete | 0.56 (0.48, 0.64) | 0.53 (0.46, 0.60) | 0.76 (0.70, 0.83) | 0.78 (0.70, 0.85) |
|  | 2 (70) | SuperLearner | 0.74 (0.66, 0.81) | 0.55 (0.45, 0.64) | 0.85 (0.78, 0.92) | 0.82 (0.70, 0.94) |
|  |  | Discrete | 0.70 (0.62, 0.78) | 0.59 (0.51, 0.67) | 0.85 (0.78, 0.92) | 0.84 (0.75, 0.92) |

*Appendix S7: Complete case analysis of random forests analysis*

For R code and output for the below results, go to *R_ELVS > RF > complete-case* on the OSF repository.

We ran complete case analysis of the random forests, excluding all participants with any missing data. Table S6 shows the there was substantial data loss in the complete case analysis, especially at waves 2-4.

1. *Number of participants classified as having “normal” (CELF-4 raw score >81 at 11 years) or “low” (≤81) language included in each wave of the random forests primary and complete case analysis*

|  | **Primary analysis** | | **Complete case** | |
| --- | --- | --- | --- | --- |
|  | **Normal** | **Low** | **Normal** | **Low** |
| Wave 1 (8 months) | 791 | 48 | 608 | 26 |
| Wave 2 (12 months) | 780 | 46 | 294 | 20 |
| Wave 3 (24 months) | 774 | 45 | 298 | 9 |
| Wave 4 (36 months) | 767 | 45 | 219 | 10 |

Table S7 shows that only a few highly ranked variables were common between the complete case and the primary analysis (blows raspberries, number of words child understands, says “circle”). We cannot ascertain whether the differences in results are due to artefacts in the imputed values in the primary analysis, or bias and lower precision in the complete case analysis due to high data missingness. We are comfortable presenting the analysis with imputed values as the primary analysis because (i) we averaged results across 300 different imputations, minimizing the effects of imputed values and (ii) the SuperLearner analysis used complete case analysis and yielded satisfactory results for waves 3 and 4. We do not discount that there could exist a better set of variables that yields higher predictive accuracy, but we have used robust methods to identify a set that yields satisfactory accuracy at 24 and 36 months.

1. *Top 10 variables per wave ranked by variable importance in the complete case analysis*

| **Variable** | **Question** | **Source** | **Variable importance** |
| --- | --- | --- | --- |
| **Wave 1** |  |  |  |
| cg12d_w1 | Have you (caregiver) completed a degree? | SDV | .000004263233 |
| sou9_w1 | Does your child blow raspberries? | SDV | .000003834049 |
| csbs22w1 | About how many of the following objects does your child use appropriately: cup, bottle, bowl, spoon, comb or brush, toothbrush, washcloth, ball, toy vehicle, toy telephone? | CSBS | .000001373391 |
| atp3_w1 | How do you think you are coping? | ATP | .000001258941 |
| nicu1a_w1 | For about how many days was this baby admitted to a neonatal intensive care unit (NICU) or special care nursery (SCN)? | SDV | .0000008869814 |
| gender | This child is a boy/girl | SDV | .0000006866953 |
| s_eco | SEIFA Economic-Resource value by post code | SEIFA | .0000006008584 |
| cgp5c_w1 | Has your partner completed a diploma/certificate? | SDV | .0000004005722 |
| csbs11w1 | Does your child wave to greet people? | CSBS | .0000003719599 |
| wg2b6w1 | Does your child dance? | CDI-WG | .0000002575107 |
| **Wave 2** |  |  |  |
| wg2c6w2 | Does your child (try to) put on a hat? | CDI-WG | .00002115942 |
| bits10_w2 | When my child looks at or touches a toy, I talk to him/her about it | BITS | .00001142029 |
| wg1d13aww2 | Understands (and says) “tickle” | CDI-WG | .000007652174 |
| wg1d7gw2 | Understands (and says) “foot” | CDI-WG | .000002318841 |
| wg1d10xw2 | Understands (and says) “tree” | CDI-WG | .0000008695652 |
| wg1d2agw2 | Understands (and says) “teddy bear” | CDI-WG | .0000005217391 |
| wg1b13w2 | Understands “Give it to mummy” | CDI-WG | .0000004637681 |
| sou2_w2 | Does your child gurgle? | SDV | .0000004057971 |
| wg1d12fw2 | Understands (and says) “hello” | CDI-WG | .0000002318841 |
| wg1d5jw2 | Understands (and says) “cheese” | CDI-WG | .0000001159420 |
| **Wave 3** |  |  |  |
| ws1a15pw3 | Says “dirty” | CDI-WS | .000005773810 |
| ws2e9w3 | Mark the sentence that sounds most like the way your child talks at the moment. “Baby blanket” or “Baby's blanket” | CDI-WS | .000002380952 |
| ws1a7kw3 | Says “finger” | CDI-WS | .000001428571 |
| atp5_w3 | My child has excessive crying | ATP | .000001369048 |
| ws1a13iw3 | Says “hi” | CDI-WS | .000001309524 |
| csbs20w3 | About how many different words or phrases does your child understand without gestures? For example, if you say "where's your tummy", "where's daddy", "give me ball", or "come here", without showing or pointing, your child will respond appropriately | CSBS | .0000008333333 |
| ws1a19rw3 | Says “on” | CDI-WS | .0000007142857 |
| ws1a7cw3 | Says “belly button” | CDI-WS | .0000004761905 |
| ws1a10abw3 | Says “swing” | CDI-WS | .0000002380952 |
| ws1a19qw3 | Says “off” | CDI-WS | .0000002380952 |
| **Wave 4** |  |  |  |
| mc3v47w4 | Says “circle” | CDI-III | .0006103968 |
| mc3s3w4 | Mark the sentence that sounds most like the way your child talks at the moment. “Coffee hot” or “That coffee hot” | CDI-III | .0001901587 |
| mc3v89w4 | Says “away” | CDI-III | .0001104762 |
| age_w4 | Age at wave 4 | SDV | .00007047619 |
| mc3s5w4 | Mark the sentence that sounds most like the way your child talks at the moment. “Don't read book” or “Don't want you read that book” | CDI-III | .00006396825 |
| mc3v69w4 | Says “empty” | CDI-III | .00005420635 |
| kes_e_w4 | In the last two weeks, about how often did you feel that everything was an effort | Kessler-K6 | .00001587302 |
| mc3v54w4 | Says “forget/forgot” | CDI-III | .00001087302 |
| mc3v43w4 | Says “farmer” | CDI-III | .000009206349 |
| mc3v30w4 | Says “stamp” | CDI-III | .000008571429 |

*Appendix S8: Univariate analysis*

For R code and output for the below results, go to *R_ELVS > ELVS uni analysis SL.Rmd*, *ELVS uni analysis.Rmd* on the OSF repository.

We ran univariate logistic regression on all variables included in the final predictor sets to allow for comparison with previous studies. To improve precision, we merged levels with no significant difference between levels and where one level had markedly fewer responses than the others. We expect the reference level to be more associated with typical language outcome. Results are presented in Table S8.

1. *Odds ratio and 95% CI of having low language outcome at 11 years (CELF-4 raw score <81). Asterisk indicates 95% CI does not cross null value of 1.*

| **Question** | **Reference** | **Odds ratio** | **95% CI** |  |
| --- | --- | --- | --- | --- |
| **Wave 1** |  |  |  |  |
| What was the last year of school your partner completed? | Year 12 |  |  |  |
| Year 10 or less |  | 2.88 | 1.43, 5.70 | * |
| Year 11 |  | 1.74 | 0.76, 3.71 |  |
| How difficult do you think your life is at present? | No/few problems or stresses |  |  |  |
| (Too) many problems or stresses |  | 4.50 | 1.79, 10.38 | * |
| Some problems or stresses |  | 1.21 | 0.62, 2.30 |  |
| I help my child learn by talking and showing him/her new things sometimes or not very often | Mostly true | 1.56 | 0.82, 2.86 |  |
| Does your child throw a ball? No | Yes | 0.60 | 0.33, 1.14 |  |
| Does your child blow raspberries? No | Yes | 2.43 | 1.34, 4.56 | * |
| When you are not paying attention to your child, does he/she try to get your attention? | Often |  |  |  |
| Not yet |  | 2.37 | 0.66, 6.84 |  |
| Sometimes |  | 1.39 | 0.75, 2.64 |  |
| Smacks lips in a "yum yum" gesture to indicate when something tastes good | Often |  |  |  |
| Not yet |  | 0.61 | 0.28, 1.47 |  |
| Sometimes |  | 0.73 | 0.3, 1.9 |  |
| I am _______ years old (caregiver) | 22-34 |  |  |  |
| ≤21 |  | 4.23 | 0.21, 29.61 |  |
| ≥35 |  | 1.04 | 0.54, 1.91 |  |
| Gestational age | 37-42 weeks |  |  |  |
| ≤36 weeks |  | 4.18 | 1.60, 9.74 | * |
| ≥43 weeks |  | 0.77 | 0.12, 2.65 |  |
| **Wave 2** |  |  |  |  |
| I am this child's stepfather or other | Biological father | 10.74 | 1.45, 57.02 | * |
| Does your child string sounds together, such as uh oh, mama, gaga, bye bye, bada? | Often |  |  |  |
| Not yet |  | 6.60 | 2.80, 14.76 | * |
| Sometimes |  | 2.19 | 1.10, 4.29 | * |
| Does your child use sounds or words to get attention or help? | Often |  |  |  |
| Not yet |  | 5.23 | 1.43, 15.42 | * |
| Sometimes |  | 1.83 | 0.95, 3.43 |  |
| In the last two weeks, about how often did you feel so sad nothing would cheer you up? Some to all of the time | None of the time | 2.41 | 1.28, 4.43 | * |
| About how many of the following objects does your child use appropriately: cup, bottle, bowl, spoon, comb or brush, toothbrush, washcloth, ball, toy vehicle, toy telephone? 0-5 | ≥6 | 2.76 | 1.50, 5.26 | * |
| Child doesn’t understand "give" | Understands | 1.27 | 0.67, 2.60 |  |
| Child doesn’t understand "don’t" | Understands | 0.89 | 0.49, 1.62 |  |
| Doesn’t seem to understand "give me a kiss" | Understands | 2.16 | 1.19, 3.97 | * |
| Child doesn’t understand "drink" | Understands | 2.97 | 1.60, 5.74 | * |
| Child doesn’t sing | Sings | 1.35 | 0.75, 2.49 |  |
| **Wave 3** |  |  |  |  |
| About how many of the following objects does your child use appropriately: cup, bottle, bowl, spoon, comb or brush, toothbrush, washcloth, ball, toy vehicle, toy telephone? 0-8 | ≥9 | 8.31 | 3.92, 16.92 | * |
| Does your child use sounds or words to get attention or help? Not yet or sometimes | Often | 5.94 | 2.51, 12.98 | * |
| Do you have any concerns about how your child behaves? A little or yes^ | No | 3.08 | 1.51, 5.96 | * |
| Doesn’t say “spoon” | Says | 5.99 | 3.24, 11.33 | * |
| About how many different words or phrases does your child understand without gestures? | ≥31 |  |  |  |
| 0-10 |  | 11.02 | 4.15, 27.56 | * |
| 11-30 |  | 4.37 | 2.24, 8.69 | * |
| Doesn’t say “face” | Says | 4.09 | 2.14, 8.37 | * |
| Doesn’t say “quack quack” | Says | 4.68 | 2.49, 8.69 | * |
| Does your child talk about objects that are not present? | Often |  |  |  |
| Not yet |  | 12.49 | 5.33, 31.72 | * |
| Sometimes |  | 3.10 | 1.40, 7.57 | * |
| Doesn’t say “teddy bear” | Says | 3.80 | 2.04, 7.01 | * |
| Doesn’t say “bubbles” | Says | 4.63 | 2.48, 8.58 | * |
| **Wave 4** |  |  |  |  |
| Mark the sentence that sounds most like the way your child talks at the moment. "This dolly big" | "This dolly big and this dolly little" | 9.77 | 4.97, 19.94 | * |
| Has your child begun to combine words yet, such as "nother biscuit" or "doggie bite?" | Often |  |  |  |
| Not yet |  | 97.93 | 13.96, 1948.55 | * |
| Sometimes |  | 10.64 | 4.49, 23.99 | * |
| Doesn’t say “circle” | Says | 8.94 | 4.67, 16.98 | * |
| Doesn’t say “accident” | Says | 7.90 | 4.21, 15.42 | * |
| Doesn’t say “forget/forgot” | Says | 8.16 | 4.35, 15.94 | * |
| I play with my child and show him/her things not very often or sometimes | Often | 0.97 | 0.46, 1.89 |  |
| How do you think you are coping? Not at all | A little to extremely well | 6.26 | 1.96, 17.08 | * |
| Child cannot answer questions | Yes | 5.91 | 3.14, 11.17 | * |
| Doesn’t say “kangaroo” | Says | 9.09 | 4.59, 17.66 | * |
| Doesn’t say “hurry” | Says | 5.87 | 3.18, 11.03 | * |

^Correction made on 5 May 2023, after first online publication: In Table S8, “Do you have any concerns about how your child uses his or her arms and legs?” has been corrected to “Do you have any concerns about how your child behaves?” in this version.

We also ran univariate logistic regression on variables we identified from previous population studies as potential predictors of language outcome (Armstrong et al., 2018; Ghassabian et al., 2014; Hammer et al., 2017; Law et al., 2012; McKean et al., 2016; Poll & Miller, 2013; Roulstone et al., 2011; Stanton-Chapman et al., 2002; Tomblin et al., 1997; Zambrana et al., 2013, 2014). Results are presented in Table S9. This analysis is not exhaustive of all variables in the ELVS sample or all possible predictors, but allows for comparison with previous studies.

1. *Odds ratios and 95% CIs of selected variables based on previous literature for predicting 11-year language outcome. Asterisk indicates 95% CI does not cross null value of 1.*

| **Question** | **Reference** | **Odds ratio** | **95% CI** |  |
| --- | --- | --- | --- | --- |
| **Demographics and family** |  |  |  |  |
| Boy | Girl | 2.00 | 1.1, 3.7 | * |
| Birth order | First |  |  |  |
| Second |  | 0.98 | 0.49, 1.91 |  |
| Third |  | 1.50 | 0.61, 3.34 |  |
| Fourth or more |  | 3.64 | 0.80, 12.07 |  |
| Mother is single or separated (8m) | Married/de facto | 1.59 | 0.25, 5.66 |  |
| Mother doesn’t have a partner currently living with child (8m) | Yes | 1.14 | 0.18, 3.95 |  |
| Mother doesn’t have a partner | Yes |  |  |  |
| At 12m |  | 3.13 | 0.71, 9.76 |  |
| At 2y |  | 1.34 | 0.21, 4.69 |  |
| At 3y |  | 2.10 | 0.61, 5.59 |  |
| **Birth** |  |  |  |  |
| Twin | Singleton | 5.05 | 1.39, 14.76 | * |
| Birthweight | 2500-4000 |  |  |  |
| <2500g |  | 2.34 | 0.67, 6.33 |  |
| >4000g |  | 0.89 | 0.26, 2.29 |  |
| Neonatal Intensive Care Unit or Special Care Nursery admission | No | 1.28 | 0.59, 2.53 |  |
| **Family history** |  |  |  |  |
| Mother was a late talker | Was not | 2.23 | 0.51, 6.71 |  |
| Father was a late talker | Was not | 1.77 | 0.28, 6.34 |  |
| Mother had speech problems | Did not | 1.51 | 0.08, 8.01 |  |
| Father had speech problems | Did not | 1.45 | 0.23, 5.12 |  |
| Mother stuttered | Did not | 4.26 | 0.63, 17.58 |  |
| Father stuttered | Did not | 0.68 | 0.04, 3.33 |  |
| Mother had reading problems | Did not | 1.63 | 0.38, 4.81 |  |
| Father had reading problems | Did not | 2.18 | 0.73, 5.35 |  |
| **Parents’ education** |  |  |  |  |
| Last year of school mother completed | Year 12 |  |  |  |
| Year 10 or less |  | 2.64 | 0.96, 6.23 |  |
| Year 11 |  | 1.27 | 0.51, 2.78 |  |
| Mother completed no other qualification | Has qualification | 1.36 | 0.32, 3.95 |  |
| Mother completed trade apprenticeship | Did not | 2.48 | 0.71, 6.67 |  |
| Mother completed diploma/certificate | Did not | 0.68 | 0.35, 1.25 |  |
| Mother completed degree | Did not | 0.37 | 0.17, 0.74 | * |
| Mother completed post-graduate qualification | Did not | 0.90 | 0.38, 1.86 |  |
| Mother completed other qualification | Did not | 1.48 | 0.35, 4.34 |  |
| Father completed no other qualification | Has qualification | 1.66 | 0.09, 8.95 |  |
| Father completed trade apprenticeship | Did not | 1.38 | 0.73, 2.54 |  |
| Father completed diploma/certificate | Did not | 1.31 | 0.65, 2.47 |  |
| Father completed degree | Did not | 0.31 | 0.12, 0.68 | * |
| Father completed post-graduate qualification | Did not | 0.49 | 0.12, 1.37 |  |
| Father completed other qualification | Did not | 1.03 | 0.16, 3.55 |  |
| **Socio-economic status** |  |  |  |  |
| Main source of income | Full-time work (one parent) |  |  |  |
| Full-time work (both parents) |  | 1.18 | 0.28, 3.42 |  |
| Part-time work, pension or other |  | 0.51 | 0.08, 1.72 |  |
| Mother or partner has a Centrelink Health Care Card | No | 1.99 | 0.94, 3.89 |  |
| SEIFA Disadvantage value by postcode | Quartile 4 |  |  |  |
| Quartile 1 |  | 1.11 | 0.45, 2.71 |  |
| Quartile 2 |  | 1.07 | 0.48, 2.47 |  |
| Quartile 3 |  | 1.12 | 0.49, 2.60 |  |
| SEIFA Advantage-Disadvantage value by postcode | Quartile 4 |  |  |  |
| Quartile 1 |  | 1.21 | 0.48, 3.00 |  |
| Quartile 2 |  | 1.70 | 0.80, 3.76 |  |
| Quartile 3 |  | 0.80 | 0.32, 1.98 |  |
| SEIFA Economic-Resource value by postcode | Quartile 4 |  |  |  |
| Quartile 1 |  | 0.81 | 0.36, 1.81 |  |
| Quartile 2 |  | 0.30 | 0.1, 0.8 | * |
| Quartile 3 |  | 0.99 | 0.48, 2.06 |  |
| SEIFA Education-Occupation value by postcode | Quartile 4 |  |  |  |
| Quartile 1 |  | 1.93 | 0.92, 4.28 |  |
| Quartile 2 |  | 0.87 | 0.33, 2.20 |  |
| Quartile 3 |  | 0.71 | 0.28, 1.74 |  |
| **Childcare** |  |  |  |  |
| Receives childcare | Does not |  |  |  |
| At 8m |  | 1.28 | 0.61, 2.49 |  |
| At 12m |  | 1.49 | 0.82, 2.72 |  |
| At 2y |  | 1.70 | 0.90, 3.36 |  |
| At 3y |  | 1.46 | 0.78, 2.88 |  |
| **Fine motor** |  |  |  |  |
| Parental concern for how child uses hands and fingers (yes or a little) | No |  |  |  |
| At 12m |  | 3.52 | 0.18, 22.47 |  |
| At 2y |  | 5.79 | 0.28, 46.25 |  |
| At 3y |  | 6.01 | 1.3, 21.0 | * |
| **Gross motor** |  |  |  |  |
| Parental concern for how child uses arms and legs (yes or a little) | No |  |  |  |
| At 8m |  | 0.99 | 0.16, 3.41 |  |
| At 12m |  | 4.12 | 1.15, 11.67 | * |
| At 2y^ |  | 0.00 | NA, 2.54e+19 |  |
| At 3y |  | 1.56 | 0.08, 8.30 |  |
| **Behaviour** |  |  |  |  |
| Parental concern about how child behaves (yes or a little) | No |  |  |  |
| At 8m |  | 1.03 | 0.24, 2.94 |  |
| At 12m |  | 1.83 | 0.73, 4.02 |  |
| At 2y |  | 3.08 | 1.51, 5.96 | * |
| At 3y |  | 2.23 | 1.14, 4.19 | * |
| Compared to other children I think my child is (8m) | Much easier |  |  |  |
| (Much) more difficult |  | 0.96 | 0.26, 2.86 |  |
| Average |  | 0.61 | 0.30, 1.29 |  |
| Easier |  | 0.34 | 0.15, 0.75 | * |
| Compared to other children I think my child is (12m) | Much easier |  |  |  |
| (Much) more difficult |  | 1.03 | 0.15, 4.48 |  |
| Average |  | 1.43 | 0.64, 3.66 |  |
| Easier |  | 0.64 | 0.25, 1.77 |  |
| Compared to other children I think my child is (2y) | Much easier |  |  |  |
| (Much) more difficult |  | 1.20 | 0.32, 4.54 |  |
| Average |  | 0.69 | 0.26, 2.13 |  |
| Easier |  | 0.68 | 0.25, 2.16 |  |
| Compared to other children I think my child is (3y) | Much easier |  |  |  |
| (Much) more difficult |  | 1.90 | 0.67, 5.86 |  |
| Average |  | 0.72 | 0.29, 2.05 |  |
| Easier |  | 0.40 | 0.14, 1.20 |  |
| Parental concerns about child learning to do things for themselves (yes or a little) | No |  |  |  |
| At 8m |  | 1.14 | 0.18, 3.94 |  |
| At 12m |  | 2.22 | 0.34, 8.15 |  |
| At 2y |  | 2.93 | 0.45, 11.18 |  |
| At 3y |  | 3.55 | 1.15, 9.04 | * |
| **Hearing loss** |  |  |  |  |
| Parental concern that child has hearing loss | No |  |  |  |
| At 8m |  | 0.86 | 0.20, 2.44 |  |
| At 12m |  | 2.20 | 0.73, 5.40 |  |
| At 2y |  | 2.14 | 0.78, 4.94 |  |
| At 3y |  | 0.91 | 0.14, 3.11 |  |
| Child notices sounds like people coming into the room or food preparation | Often |  |  |  |
| Not yet |  | 1.48 | 0.43, 3.96 |  |
| Sometimes |  | 0.67 | 0.32, 1.32 |  |
| Result of hearing test (8m) | No test or normal hearing |  |  |  |
| Poor hearing in both ears |  | 3.19 | 0.48, 12.46 |  |
| Poor hearing in one ear |  | 1.15 | 0.49, 2.41 |  |
| Result of hearing test (12m) | No test or normal hearing |  |  |  |
| Poor hearing in both ears |  | 3.78 | 0.57, 15.06 |  |
| Poor hearing in one ear |  | 1.35 | 0.54, 2.95 |  |
| Result of hearing test was poor hearing in one or both ears (2y) | No test or normal hearing | 1.68 | 0.26, 5.99 |  |
| Result of hearing test was poor hearing in one or both ears (3y) | No test or normal hearing | 1.99 | 0.11, 10.96 |  |
| **Gestures** |  |  |  |  |
| Extends arm to show you something they are holding at 8m (CDI-WG) | Often |  |  |  |
| Not yet |  | 0.82 | 0.36, 2.03 |  |
| Sometimes |  | 0.98 | 0.44, 2.40 |  |
| Extends arm to show you something they are holding at 12m (CDI-WG) | Often |  |  |  |
| Not yet |  | 1.92 | 0.44, 5.88 |  |
| Sometimes |  | 1.68 | 0.89, 3.12 |  |
| Points at 8m | Often |  |  |  |
| Not yet |  | 1.46 | 0.30, 26.32 |  |
| Sometimes |  | 2.72 | 0.48, 51.35 |  |
| Points at 12m | Often |  |  |  |
| Not yet |  | 2.44 | 1.24, 4.88 | * |
| Sometimes |  | 1.17 | 0.50, 2.58 |  |
| **Speech and language** |  |  |  |  |
| Parental concern for speech sounds at 8m (yes or a little) | No | 1.81 | 0.42, 5.36 |  |
| Parental concern for speech sounds at 12m (yes or a little) | No | 1.90 | 0.55, 5.02 |  |
| Parental concern for speech sounds at 2y | No |  |  |  |
| “A little” |  | 2.31 | 1.01, 4.83 | * |
| “Yes” |  | 2.94 | 0.83, 8.06 |  |
| Parental concern for speech sounds at 3y | No |  |  |  |
| “A little” |  | 1.30 | 0.54, 2.79 |  |
| “Yes” |  | 5.37 | 2.15, 12.29 | * |
| Parental concern for how much child understands at 8m (yes or a little) | No | 2.84 | 0.65, 8.80 |  |
| Parental concern for how much child understands at 12m (yes or a little) | No | 5.01 | 1.6, 13.2 | * |
| Parental concern for how much child understands at 2y (yes or a little) | No | 2.33 | 0.36, 8.62 |  |
| Parental concern for how much child understands at 2y (yes or a little) | No | 3.13 | 0.71, 9.80 |  |
| Strings sounds together at 9m | Often |  |  |  |
| Not yet |  | 0.97 | 0.45, 1.99 |  |
| Sometimes |  | 0.77 | 0.37, 1.51 |  |
| Words or phrases understood at 8m (CSBS) | ≥4 |  |  |  |
| None |  | 2.47 | 0.93, 8.53 |  |
| 1-3 |  | 1.92 | 0.71, 6.70 |  |
| Words or phrases understood at 12m (CSBS) | ≥4 |  |  |  |
| None |  | 3.91 | 1.62, 8.83 | * |
| 1-3 |  | 1.76 | 0.90, 3.43 |  |
| Words or phrases understood at 2y (CSBS) | ≥31 |  |  |  |
| ≤10 |  | 11.02 | 4.15, 27.56 | * |
| 11-30 |  | 4.37 | 2.24, 8.69 | * |
| Words spoken at 8m (CSBS) | ≥4 |  |  |  |
| None |  | 0.81 | 0.27, 3.46 |  |
| 1-3 |  | 1.11 | 0.34, 4.96 |  |
| Words spoken at 12m (CSBS) | ≥11 |  |  |  |
| None |  | 2.59 | 0.50, 47.51 |  |
| 1-3 |  | 1.42 | 0.27, 26.05 |  |
| 4-10 |  | 1.34 | 0.25, 24.84 |  |
| ≤30 words spoken at 2y (CSBS) | ≥31 | 4.01 | 2.18, 7.56 | * |
| Not combining words at 8m | Sometimes or often | 1.16 | 0.23, 21.01 |  |
| Not combining words at 12m | Sometimes or often | 1.11 | 0.43, 3.76 |  |
| Combining words at 2y (CSBS) | Often |  |  |  |
| Not yet |  | 2.83 | 1.17, 6.93 | * |
| Sometimes |  | 0.43 | 0.21, 0.94 | * |
| Combining words at 2y (CDI) | Often |  |  |  |
| Not yet |  | 5.88 | 2.63, 13.51 | * |
| Sometimes |  | 2.87 | 1.36, 6.32 | * |
| Not or sometimes combining words at 3y (CDI) | Often | 14.28 | 6.60, 30.29 | * |
| Child talks about past events or people who are not present at 2y | Often |  |  |  |
| Not yet |  | 6.51 | 2.84, 17.62 | * |
| Sometimes |  | 1.07 | 0.38, 3.22 |  |
| Child talks about something that's going to happen in the future at 2y | Often |  |  |  |
| Not yet |  | 11.82 | 4.03, 50.43 | * |
| Sometimes |  | 4.07 | 1.37, 17.45 | * |
| Child understands if you ask for something that is not in the room at 2y | Often |  |  |  |
| Not yet |  | 5.99 | 0.88, 25.34 |  |
| Sometimes |  | 3.18 | 1.61, 6.09 | * |
| Child picks up or point to an object and name an absent person to whom the object belongs at 2y | Often |  |  |  |
| Not yet |  | 5.05 | 2.00, 11.74 | * |
| Sometimes |  | 2.32 | 1.17, 4.52 | * |
| Child adds an "s" to words to talk about more than one thing at 2y | Often |  |  |  |
| Not yet |  | 2.95 | 1.27, 8.05 | * |
| Sometimes |  | 1.18 | 0.46, 3.40 |  |
| Child adds an "s" to words to talk about ownership at 2y | Often |  |  |  |
| Not yet |  | 3.61 | 1.64, 8.77 | * |
| Sometimes |  | 2.11 | 0.89, 5.35 |  |
| Child adds an "ing" to words to talk about activities at 2y | Often |  |  |  |
| Not yet |  | 4.98 | 1.95, 16.84 | * |
| Sometimes |  | 1.38 | 0.39, 5.47 |  |
| Child adds “ed” to words to talk about things that happened in the past at 2y | Often |  |  |  |
| Not yet |  | 2.81 | 0.59, 50.47 |  |
| Sometimes |  | 2.05 | 0.35, 38.92 |  |
| Child doesn’t understand the concept of “one” at 3y | Yes | 6.74 | 2.33, 17.26 | * |
| Child doesn’t ask questions with more than one word that begin "what" or "where" at 3y | Yes | 8.09 | 3.48, 17.68 | * |
| Child doesn’t ask questions with more than one word that begin "why" or "how" at 3y | Yes | 3.84 | 2.01, 7.20 | * |
| Child doesn’t give reasons for things, using the word "because" at 3y | Yes | 4.08 | 2.22, 7.69 | * |
| If you asked your child "What is a horse?", they could not answer "an animal" at 3y | Yes | 10.24 | 3.68, 42.57 | * |
| Child cannot name simple shapes with the words "circle", "square" and "triangle" at 3y | Yes | 3.34 | 1.63, 6.51 | * |
| Child does not talk about things that "could" or "might happen at 3y | Yes | 4.32 | 2.35, 8.09 | * |
| Child does not ever ask what a particular word means at 3y | Yes | 6.18 | 2.79, 16.41 | * |
| Child could not tell you which of two objects is larger if they were not present at 3y | Yes | 3.09 | 1.68, 5.80 | * |
| Child does not use -est words such as "biggest" and "strongest" | Yes | 4.43 | 2.20, 9.91 | * |
| **Parent-child interactions** |  |  |  |  |
| When child looks at or touches a toy, parent talks about it at 8m | Often |  |  |  |
| Not very true |  | 1.58 | 0.56, 3.91 |  |
| Sometimes true |  | 0.84 | 0.45, 1.59 |  |
| When child looks at or touches a toy, parent talks about it at 12m | Often |  |  |  |
| Not very often |  | 5.07 | 1.94, 12.36 | * |
| Sometimes |  | 1.41 | 0.73, 2.77 |  |
| When child looks at or touches a toy, parent talks about it “not very often” or “sometimes” at 2y | Often | 2.29 | 1.24, 4.37 | * |
| When child looks at or touches a toy, parent talks about it “not very often” or “sometimes” at 3y | Often | 1.74 | 0.92, 3.48 |  |
| Parent looks at or reads children's books to child | Often |  |  |  |
| Not very true |  | 1.94 | 0.86, 4.42 |  |
| Sometimes true |  | 1.57 | 0.78, 3.32 |  |
| Number of children’s books in the home | ≥31 |  |  |  |
| ≤20 |  | 3.27 | 1.51, 6.76 | * |
| 21-30 |  | 2.86 | 1.33, 5.89 | * |

^Correction made on 5 May 2023, after first online publication: In Table S9, “Do you have any concerns about how your child uses his or her arms and legs?” at 2 years has been added in this version.

*Appendix S9: Multilingual subgroup analysis*

For R code and output for the below results, go to *R_ELVS > SL > eleven-year15-multi, seven-year15-multi* on the OSF repository.

We attempted to assess the classification accuracy of the preferred model in the subgroup of participants whose parents reported speaking an additional language to English at home or to the child at waves 1, 3 or 4 (these questions were not asked in wave 2). We would like to know if this set of questions is suitable to use with multilingual children, or if a separate set of questions would be more appropriate.

The subgroup of multilingual children who had an 11-year outcome measure consisted of only 39 participants. With the cut-off of 1.5SD below the mean, 3 of these children were in the low language group. Even fewer participants were included in the models once we removed participants with missing data. Wave 1 models failed to converge. Table S10 shows that the models that successfully ran yielded very large 95% CIs (sensitivity 95% CIs sometimes ranged from 0.01-0.99). This indicates very low precision and that any results suggesting high accuracy may be spurious.

1. *SuperLearner accuracy (AUC), sensitivity and specificity by wave in the multilingual subgroup of participants. Large 95% CIs for sensitivity indicate low precision due to small number of children with low language ability in this subgroup (n=3)*

|  |  | **AUC** | **Sensitivity** | **Specificity** |
| --- | --- | --- | --- | --- |
| Wave 2 (12 months) | SL | 0.47 (0.28, 0.66) | 0.50 (0.01, 0.99) | 0.52 (0.34, 0.69) |
|  | Discrete | 0.71 (0.52, 0.91) | 0.50 (0.01, 0.99) | 0.64 (0.45, 0.80) |
| Wave 3 (24 months) | SL | 0.54 (0.34, 0.74) | 0.67 (0.09, 0.99) | 0.63 (0.45, 0.79) |
|  | Discrete | 0.81 (0.54, 1.00) | 1.00 (0.29, 1.00) | 0.63 (0.45, 0.79) |
| Wave 4 (36 months) | SL | 0.63 (0.30, 0.96) | 0.67 (0.09, 0.99) | 0.70 (0.50, 0.86) |
|  | Discrete | 0.67 (0.44, 0.90) | 0.67 (0.09, 0.99) | 0.74 (0.54, 0.89) |

We examined the AUC of the models using the 7-year language outcome (1.5SD below the mean cut-off), which consisted of 59 participants, 6 of whom were in the low language group. Table S11 shows that results yielded low accuracy and wide 95% CIs, like in the 11-year subgroup.

1. *SuperLearner accuracy (AUC) by wave in the multilingual subgroup of participants with the 7-year language outcome. Large 95% CIs for sensitivity indicate low precision due to small number of children with low language ability in this subgroup (n=6)*

|  |  | **AUC** |
| --- | --- | --- |
| Wave 2 (12 months) | SL | 0.23 (0.09, 0.37) |
|  | Discrete | 0.31 (0.17, 0.45) |
| Wave 3 (24 months) | SL | 0.54 (0.27, 0.82) |
|  | Discrete | 0.43 (0.21, 0.64) |
| Wave 4 (36 months) | SL | 0.38 (0.14, 0.63) |
|  | Discrete | 0.49 (0.18, 0.80) |

We cannot make any conclusions based on our results as to the accuracy of these sets of questions for predicting low language outcome in multilingual children. Future research should target a larger group of multilingual children to investigate the accuracy of these questions or identify an accurate set of measures tailored to multilingual children.

*Appendix S10: Exploratory analysis of predictors mapping to LSAC variables*

For R code and output for the below results, go to *R_ELVS > SL > lsac* on the OSF repository.

We will replicate the current study using data from Growing Up in Australia: The Longitudinal Study of Australian Children (LSAC) (Gasparini et al., 2022; Sanson & Johnstone, 2004). We ran SuperLearner models using only variables ranked highly in the Aim 1 analysis that were also collected in LSAC. This included (i) 8 predictors from ELVS waves 1 and 2 in one model to map to LSAC’s wave 1 (ages 0-1), (ii) 8 predictors from ELVS waves 3 and 4 in a second model to map to LSAC’s wave 2 (ages 2-3), and (iii) those 16 predictors combined in one model. We removed one variable at a time in ascending order of variable importance to identify which model had the best accuracy.

The variables included in each of the best-fitting models are presented in Table S12 (waves 1-2), Table S13 (waves 3-4) and Table S14 (waves 1-4).

1. *The 8 variables in the best-fitting model from ELVS waves 1-2 that correspond to LSAC wave 1 variables*

| **ELVS variable** | **Question** | **Options** | **Source** | **LSAC variable** |
| --- | --- | --- | --- | --- |
| csbs15w2 | Does your child string sounds together, such as uh oh, mama, gaga, bye bye, bada? | Not Yet  Sometimes  Often | CSBS | alc01b1b |
| csbs14w2 | Does your child use sounds or words to get attention or help? | Not Yet  Sometimes  Often | CSBS | alc01b1a |
| kes_a_w2 | In the last two weeks, about how often did you feel so sad nothing would cheer you up? | None of the time  A little of the time  Some of the time  Most of the time  All of the time | Kessler-K6 | ahs24a5 |
| cgp4_w1 | What was the last year of school your partner completed? (Tick one box) | Year 10 or less  Year 11  Year 12 | SDV | afd08b1 |
| atp2_w1 | How difficult do you think your life is at present? (tick one box) | No problems or stresses  Few problems or stresses  Some problems or stresses  Many problems or stresses  Too many problems or stresses | ATP | ahs26a1 |
| csbs22w2 | About how many of the following objects does your child use appropriately: cup, bottle, bowl, spoon, comb or brush, toothbrush, washcloth, ball, toy vehicle, toy telephone? | None  1-2  3-4  5-8  over 8 | CSBS | alc01c2b |
| csbs6w1 | When you are not paying attention to your child, does he/she try to get your attention? | Not Yet  Sometimes  Often | CSBS | alc01a2b |
| cg1_w1 | I am _______ years old (caregiver) | Numeric | SDV | af03m2 |

1. *The 6 variables in the best-fitting model from ELVS waves 3-4 that correspond to LSAC wave 2 variables*

| **ELVS variable** | **Question** | **Options** | **Source** | **LSAC variable** |
| --- | --- | --- | --- | --- |
| mc3s10w4 | Mark the sentence that sounds MOST like the way your child talks at the moment. If your child is saying sentences even longer or more complicated than the two provided, mark the second one. | This dolly big This dolly big and this dolly little | CDI-III | blc07b10 |
| mc3v47w4 | “Circle” | Says  Not yet | CDI-III | blc07a46 |
| mc3v46w4 | “Accident” | Says  Not yet | CDI-III | blc07a45 |
| peds6_w3 | Do you have any concerns about how your child behaves?^ | No  Yes  A little | PEDS | bgd01a4 |
| mc3v3w4 | “Kangaroo” | Says  Not yet | CDI-III | blc07a3 |
| mc3v54w4 | “Forget/forgot” | Says  Not yet | CDI-III | blc07a53 |

^Correction made on 5 May 2023, after first online publication: In Table S13, “Do you have any concerns about how your child uses his or her arms and legs?” has been corrected to “Do you have any concerns about how your child behaves?” in this version.

1. *The 11 variables in the best-fitting model from ELVS waves 1-4 that correspond to LSAC wave 1-2 variables*

| **ELVS variable** | **Question** | **Options** | **Source** | **LSAC variable** |
| --- | --- | --- | --- | --- |
| mc3s10w4 | Mark the sentence that sounds MOST like the way your child talks at the moment. If your child is saying sentences even longer or more complicated than the two provided, mark the second one. | This dolly big This dolly big and this dolly little | CDI-III | blc07b10 |
| mc3v47w4 | “Circle” | Says  Not yet | CDI-III | blc07a46 |
| mc3v46w4 | “Accident” | Says  Not yet | CDI-III | blc07a45 |
| csbs15w2 | Does your child string sounds together, such as uh oh, mama, gaga, bye bye, bada? | Not Yet  Sometimes  Often | CSBS | alc01b1b |
| peds6_w3 | Do you have any concerns about how your child behaves?^ | No  Yes  A little | PEDS | bgd01a4 |
| mc3v3w4 | “Kangaroo” | Says  Not yet | CDI-III | blc07a3 |
| csbs14w2 | Does your child use sounds or words to get attention or help? | Not Yet  Sometimes  Often | CSBS | alc01b1a |
| kes_a_w2 | In the last two weeks, about how often did you feel so sad nothing would cheer you up? | None of the time  A little of the time  Some of the time  Most of the time  All of the time | Kessler-K6 | ahs24a5 |
| cgp4_w1 | What was the last year of school your partner completed? (Tick one box) | Year 10 or less  Year 11  Year 12 | SDV | afd08b1 |
| mc3v54w4 | “Forget/forgot” | Says  Not yet | CDI-III | blc07a53 |
| bits1_w4 | I play with my child and show him/her things | Not very often  Sometimes  Often | BITS | bre02a1b |

^Correction made on 5 May 2023, after first online publication: In Table S14, “Do you have any concerns about how your child uses his or her arms and legs?” has been corrected to “Do you have any concerns about how your child behaves?” in this version.

Results are presented in Table S15. All best-fitting models were the discrete winners (rather than SuperLearner).

1. *Accuracy (95% CIs) of the best-fitting models using ELVS variables that correspond to LSAC variables*

|  | **AUC** | **Sensitivity** | **Specificity** |
| --- | --- | --- | --- |
| Waves 1-2 (8 & 12m) | .67 (.58, .76) | .64 (.48, .78) | .67 (.63, .70) |
| Waves 3-4 (24 & 36m) | .78 (.68, .88) | .75 (.58, .88) | .81 (.78, .83) |
| Waves 1-4 | .82 (.74, .89) | .75 (.57, .89) | .73 (.70, .77) |

Like in the primary analysis, the model using variables from Waves 1 and 2 did not have satisfactory accuracy. The Wave 3-4 model had similar accuracy to the best-fitting model in the primary analysis (Wave 4, which had AUC: .79 [.70, .88], sensitivity .75 [.58, .88] and specificity .84 [.81, .87]) but specificity was slightly lower. AUC improved by combining variables between waves, but this did not improve sensitivity or specificity.

References

Armstrong, R., Symons, M., Scott, J. G., Arnott, W. L., Copland, D. A., McMahon, K. L., & Whitehouse, A. J. O. (2018). Predicting language difficulties in middle childhood from early developmental milestones: A comparison of traditional regression and machine learning techniques. *Journal of Speech, Language, and Hearing Research*, *61*(8), 1926–1944. https://doi.org/10.1044/2018_JSLHR-L-17-0210

Australian Bureau of Statistics. (2018, March 27). *Socio-Economic Indexes for Areas*. Australian Bureau of Statistics. https://www.abs.gov.au/websitedbs/censushome.nsf/home/seifa

Boehmke, B., & Greenwell, B. (2020). *Hands-On Machine Learning with R*. Chapman and Hall/CRC. https://bradleyboehmke.github.io/HOML/

Breiman, L. (2001). Random Forests. *Machine Learning*, *45*, 5–32. https://doi.org/10.1023/A:1010933404324

Chen, T., & Guestrin, C. (2016). XGBoost: A scalable tree boosting system. *Proceedings of the 22nd ACM SIGKDD International Conference on Knowledge Discovery and Data Mining*, 785–794. https://doi.org/10.1145/2939672.2939785

Chen, T., He, T., & Benesty, M. (n.d.). *XGBoost presentation*. Retrieved March 31, 2022, from https://cran.r-project.org/web/packages/xgboost/vignettes/xgboostPresentation.html

Chen, T., He, T., Benesty, M., Khotilovich, V., Tang, Y., Cho, H., Chen, K., Mitchell, R., Cano, I., Zhou, T., Li, M., Xie, J., Lin, M., Geng, Y., Li, Y., & Yuan, J. (2022). *xgboost: Extreme Gradient Boosting* (R package version 1.5.2.1). https://cran.r-project.org/web/packages/xgboost/index.html

Dale, P. S. (2007). *MacArthur-Bates Communicative Development Inventories, Third Edition*.

Dashti, S. G., Lee, K. J., Simpson, J. A., White, I. R., Carlin, J. B., & Moreno-Betancur, M. (2021). *Handling missing data when estimating causal effects with Targeted Maximum Likelihood Estimation*. arXiv. https://doi.org/10.48550/arXiv.2112.05274

de Aguiar, V., Bastiaanse, R., & Miceli, G. (2016). Improving production of treated and untreated verbs in aphasia: A meta-analysis. *Frontiers in Human Neuroscience*, *10*. https://doi.org/10.3389/fnhum.2016.00468

Fenson, L., Dale, P. S., Reznick, S., Thal, D., Bates, E., Hartung, J., Pethick, S., & Reilly, J. (1993). *MacArthur Communicative Development Inventories: User’s guide and manual*. Singular Publishing Group.

Friedman, J., Hastie, T., & Tibshirani, R. (2010). Regularization Paths for Generalized Linear Models via Coordinate Descent. *Journal of Statistical Software*, *33*(1). https://doi.org/10.18637/jss.v033.i01

Gasparini, L., Shepherd, D. A., Wang, J., Wake, M., & Morgan, A. T. (2022). *Estimating the accuracy of early life predictors of 11-year language outcome: A replication study* [Preregistration]. https://osf.io/jk32c/

Ghassabian, A., Rescorla, L., Henrichs, J., Jaddoe, V. W., Verhulst, F. C., & Tiemeier, H. (2014). Early lexical development and risk of verbal and nonverbal cognitive delay at school age. *Acta Paediatrica*, *103*(1), 70–80. https://doi.org/10.1111/apa.12449

Glascoe, F. P. (1999). Using Parents’ Concerns to Detect and Address Developmental and Behavioral Problems. *Journal for Specialists in Pediatric Nursing*, *4*(1), 24–35. https://doi.org/10.1111/j.1744-6155.1999.tb00077.x

Glascoe, F. P., & Brigance, A. (2002). *Brigance Infant and Toddler Screen: Parent-Child Interactions Form*. Curriculum Associates.

Gremmell, D. (2018, February 20). *Ensemble Learning in R with SuperLearner*. Datacamp. https://www.datacamp.com/tutorial/ensemble-r-machine-learning

Hammer, C. S., Morgan, P., Farkas, G., Hillemeier, M., Bitetti, D., & Maczuga, S. (2017). Late talkers: A population-based study of risk factors and school readiness consequences. *Journal of Speech, Language, and Hearing Research*, *60*(3), 607–626. https://doi.org/10.1044/2016_JSLHR-L-15-0417

Hastie, T., Qian, J., & Tay, K. (2021). *An Introduction to glmnet*. https://glmnet.stanford.edu/articles/glmnet.html

Hoffman, K. (2019, September 10). *Become a Superlearner*. New York, NY, NYC R Ladies September Meetup. https://www.khstats.com/talk/superlearner/

Hothorn, T. (2005). Survival ensembles. *Biostatistics*, *7*(3), 355–373. https://doi.org/10.1093/biostatistics/kxj011

Kapelner, A., & Bleich, J. (2016). **bartMachine**: Machine Learning with Bayesian Additive Regression Trees. *Journal of Statistical Software*, *70*(4). https://doi.org/10.18637/jss.v070.i04

Kennedy, C. (2017, March 16). *Guide to SuperLearner* [R vignette]. Guide to SuperLearner. https://cran.r-project.org/web/packages/SuperLearner/vignettes/Guide-to-SuperLearner.html

Kessler, R., & Mroczek, D. (1994). *Final Version of Our Non-Specific Psychological Distress Scale [Memorandum]*. Institute for Social Research.

Körting, T. S. (Director). (2014, April 5). *How Random Forest algorithm works*. https://youtu.be/loNcrMjYh64

Kuhn, M. (2021). *caret: Classification and Regression Training* (R package version 6.0-90). https://CRAN.R-project.org/package=caret

Law, J., Rush, R., Anandan, C., Cox, M., & Wood, R. (2012). Predicting Language Change Between 3 and 5 Years and Its Implications for Early Identification. *Pediatrics*, *130*(1), e132–e137. https://doi.org/10.1542/peds.2011-1673

LeDell, E., Petersen, M., & van der Laan, M. (2014). *CvAUC: Cross-Validated Area Under the ROC Curve Confidence Intervals* (R package version 1.1.0). https://CRAN.R-project.org/package=cvAUC

Liaw, A., & Wiener, M. (2002). Classiﬁcation and regression by randomForest. *R News*, *2*(3), 18–22.

Mamun, O. (2021, May 3). *A Primer to Bayesian Additive Regression Tree with R: A Bayesian approach to traditional ensemble based algorithm*. Towards Data Science. https://towardsdatascience.com/a-primer-to-bayesian-additive-regression-tree-with-r-b9d0dbf704d

McKean, C., Law, J., Mensah, F., Cini, E., Eadie, P., Frazer, K., & Reilly, S. (2016). Predicting meaningful differences in school-entry language skills from child and family factors measured at 12 months of age. *International Journal of Early Childhood*, *48*(3), 329–351. https://doi.org/10.1007/s13158-016-0174-0

Meyer, D., Dimitriadou, E., Hornik, K., Weingessel, A., Leisch, F., Chang, C.-C., & Lin, C.-C. (n.d.). *Misc Functions of the Department of Statistics, Probability Theory Group (Formerly: E1071)* (R package version 1.7-9). Retrieved March 31, 2022, from https://cran.r-project.org/web/packages/e1071/e1071.pdf

Milborrow, S. (2021a). *Notes on the earth package*. http://www.milbo.org/doc/earth-notes.pdf

Milborrow, S. (2021b). *earth: Multivariate Adaptive Regression Splines* (R package version 5.3.1). https://CRAN.R-project.org/package=earth

Mouselimis, L. (2021). *Kernel k Nearest Neighbors* (R package version 1.1.4). https://cran.r-project.org/web/packages/KernelKnn/KernelKnn.pdf

Phillips, R. V., van der Laan, M. J., Lee, H., & Gruber, S. (2022). *Practical considerations for specifying a super learner* (p. 13) [Preprint]. https://doi.org/10.48550/arXiv.2204.06139

Poll, G. H., & Miller, C. A. (2013). Late talking, typical talking, and weak language skills at middle childhood. *Learning and Individual Differences*, *26*, 177–184. https://doi.org/10.1016/j.lindif.2013.01.008

Prior, M., Sanson, A., Smart, D., & Oberklaid, F. (2000). *Pathways from Infancy to Adolescence: Australian Temperament Project 1983-2000* [Data set]. Australian Institute of Family Studies. https://doi.org/10.1037/e567532013-001

Raven, J. C. (1997). *Mill Hill Vocabulary Scale*. JC Raven Ltd.

Roulstone, S., Law, J., Rush, R., Clegg, J., & Peters, T. (2011). *Investigating the role of language in children’s early educational outcomes* [Data set]. Department for Education. https://doi.org/10.1037/e603032011-001

Sanson, A., & Johnstone, R. (2004). “Growing Up in Australia” takes its first steps. *Family Matters*, *67*, 46–53.

Sewell, J., Oberklaid, F., Prior, M., Sanson, A., & Kyrios, M. (1988). Temperament in Australian toddlers. *Journal of Paediatrics and Child Health*, *24*(6), 343–345. https://doi.org/10.1111/j.1440-1754.1988.tb01385.x

Shonkoff, J. P. (2010). Building a New Biodevelopmental Framework to Guide the Future of Early Childhood Policy. *Child Development*, *81*(1), 357–367. https://doi.org/10.1111/j.1467-8624.2009.01399.x

Stanton-Chapman, T. L., Chapman, D. A., Bainbridge, N. L., & Scott, K. G. (2002). Identification of early risk factors for language impairment. *Research in Developmental Disabilities*, *23*(6), 390–405. https://doi.org/10.1016/S0891-4222(02)00141-5

Starmer, J. (Director). (2018, February 6). *StatQuest: Random Forests Part 1—Building, Using and Evaluating*. https://youtu.be/J4Wdy0Wc_xQ

Starmer, J. (Director). (2020, January 16). *StatQuest: Random Forests Part 2: Missing data and clustering*. https://youtu.be/sQ870aTKqiM

Starmer, J. (2022). *StatQuest with Josh Starmer*. YouTube. https://www.youtube.com/user/joshstarmer?app=desktop

Stevenson, M., & Sergeant, E. (2021). *EpiR: Tools for the Analysis of Epidemiological Data* (R package version 2.0.39). https://CRAN.R-project.org/package=epiR

Strobl, C., Boulesteix, A.-L., Zeileis, A., & Hothorn, T. (2007). Bias in random forest variable importance measures: Illustrations, sources and a solution. *BMC Bioinformatics*, *8*(1), 25. https://doi.org/10.1186/1471-2105-8-25

Strobl, C., Hothorn, T., & Zeileis, A. (2009). Party on! A new, conditional variable-importance measure for random forests available in the party package. *The R Journal*, *1*(2), 14–17.

Tagliamonte, S. A., & Baayen, R. H. (2012). Models, forests, and trees of York English: *Was/were* variation as a case study for statistical practice. *Language Variation and Change*, *24*(2), 135–178. https://doi.org/10.1017/S0954394512000129

Tomblin, J. B., Smith, E., & Zhang, X. (1997). Epidemiology of specific language impairment: Prenatal and perinatal risk factors. *Journal of Communication Disorders*, *30*(4), 325–344. https://doi.org/10.1016/S0021-9924(97)00015-4

Udacity (Director). (2016, June 7). *Bootstrap aggregating bagging*. https://youtu.be/2Mg8QD0F1dQ

van der Laan, M. J., Polley, E. C., & Hubbard, A. E. (2007). Super Learner. *Statistical Applications in Genetics and Molecular Biology*, *6*(1). https://doi.org/10.2202/1544-6115.1309

Varni, J. W., Burwinkle, T. M., Seid, M., & Skarr, D. (2003). The PedsQL 4.0 as a pediatric population health measure: Feasibility, reliability, and validity. *Ambulatory Pediatrics*, *3*(6), 329–341.

Venables, W. N., & Ripley, B. D. (2002). *Modern Applied Statistics with S* (4th ed.). Springer. http://www.stats.ox.ac.uk/pub/MASS4

Wetherby, A., & Prizant, B. (2002). *Communication and Symbolic Behaviour Scales*. Paul H. Brookes.

Zambrana, I. M., Pons, F., Eadie, P., & Ystrom, E. (2014). Trajectories of language delay from age 3 to 5: Persistence, recovery and late onset. *International Journal of Language & Communication Disorders*, *49*(3), 304–316. https://doi.org/10.1111/1460-6984.12073

Zambrana, I. M., Ystrom, E., Schjølberg, S., & Pons, F. (2013). Action Imitation at 1½ Years Is Better Than Pointing Gesture in Predicting Late Development of Language Production at 3 Years of Age. *Child Development*, *84*(2), 560–573. https://doi.org/10.1111/j.1467-8624.2012.01872.x
